# The Hospital Nursing Supplement

**Published:** 1892-12-03

**Authors:** 


### "The Hospital" Nursing Mirror.

Being the Extra Nursing Supplement of "The Hospital" Newspaper.

[Contributions for this Supplement should be addressed to the Editor, The Hospital, 140, Strand, London, W.C., and should have the word "Nursing" plainly written in left-hand top corner of the envelope.]

### En Passant.

LADY DOCTORS.—It is with much pleasure that we hear that Miss Maida Sturge, M.B.Lond., has been appointed one of the assistant resident medical officers at he Stamford Hill Fever Hospital, under the Metropolitan Asylums Board. Miss Sturge is a distinguished student of the London School of Medicine for Women, and was in June last awarded the Helen Pridaux prize.

THE TRAINED NURSES' CLUB.—The sale of work held, by kind permission of Dr. and Mrs. F. R. Humphreys, at their house at South Hampstead, on Saturday last, was in aid of this club, and a brisk business was carried on. There were lady palmists and amateur theatricals, and charming recitations by Mrs. Moon Parker to amuse the many buyers who came to the sale, and the whole was well-managed. The proceeds are to go to improving this club generally, and we believe that some structural alterations are contemplated at the club rooms in Buckingham Street, as the present lecture rooms have proved lately quite inadequate to hold the large audiences which have gathered to the medical lectures.

AN INTERNATIONAL NURSING CONGRESS.—Some misapprehension seems to prevail as to the origin of the proposal to hold an International Congress on Nursing at Chicago next year. Communications have been sent to the Press, claiming that the idea emanated from an English source. Our patriotism would have made us rejoice had this been the case, but justice compels us to give credit to the United States, where the idea originated, and was put into practical shape by the Department of Moral and Social Reform Congresses so long ago as July 25th, 1892. In a circular (No. 1) of that date, issued from Chicago, it is stated that an International Congress of Charities, Correction, and Philanthropy will be held in Chicago during the week commencing June 12th, 1893. The Congress was organised in nine sections, section 4 being "on the hospital care of the sick, the training of nurses, dispensary work, and first-aid to the injured." Other sections include the prevention and relief of pauperism, the care of neglected, abandoned, and dependent children; the care and treatment of juvenile delinquents; the commitment, attention, care, and treatment of the insane; the custodial care, and the training and development of idiots and feeble-minded children; the prevention and repression of crime, and the punishment and reformation of criminals; the organisation and affiliation of charities in cities, towns, and villages; and the introduction of sociology as a special topic of investigation and teaching in the curriculum of institutions of learning. It is desirable to give the whole of the sections so that the public may grasp the wide scope and important bearings of the Chicago International Congresses of Charities, Correction, and Philanthropy to be held in June next. Hospital care and the training of nurses only represents one section, though an important one, of the ground covered by the International Congresses, which are under the direction of a committee of organisation, which includes that very able philanthropist, the Rev. F. H. Wines, Mr. John G. Shortall, and Mrs. J. M. Flower. Mr. N. S. Rosenau is the Secretary, and all communications should be addressed to him, "World's Congress Headquarters, Chicago, Illinois." The appointment of Dr. John S. Billings, of Washington, the most eminent authority in the United States, as Chairman of the Section on Hospitals, Nursing, and the Care of the Sick, would give world-wide satisfaction, and ensure many practical results of high value, which may be regarded as otherwise unattainable.

FESTIVITIES IN THE EMERALD ISLE.—The City of Dublin Hospital Nurses had an "at home" last week, to which numbers of guests came, many of whom were medical men. Mrs. Armytage-Power and other ladies provided music, and it was altogether a very pleasant gathering, to which much interest was added by the presentation of a watch, which was made to Miss Bruce in recognition of her unselfish devotion to the sick poor of North Tipperary while staying there on her summer holiday.

PRIVATE NURSES' ASSOCIATION AND CLUB, DUBLIN.—This undertaking for nurses working on their own account and receiving their own fees is fairly on its way, and the home at 97, Lower Leeson Street is ready, and some of the nurses are in it. Nurses working for themselves pay only when in the home, and a registration book is kept for non-resident nurses; those who live in the home are required to give references as to their personal character from a well-known doctor and a lady, apart from their training references. The club is open to nurses attached to other institutions, and there they can see the newspapers and have tea, while nurses who are not resident in Dublin can become country members and can use the club-room, and have board and lodging at the rate charged to resident nurses. Lady medical students can also be boarded.

SHORT ITEMS.—We acknowledge with many thanks one guinea from Mrs. Creighton Hale towards our "Endowed Bed for a Sick Nurse."—Sutton Surrey now possesses a nurses' institute in St. James' Road. Miss King, formerly of the Army Nursing Service, and Miss A. F. Van are the Lady Superintendents; Miss King holds the Royal Red Cross. The nurses are to receive a percentage on their earnings, in addition to salary.—The Countess of Carlisle has kindly promised to give £70 a year, for ten years, to provide an extra nurse for the Denton Holme Association, Carlisle, who is to work in the Caldewgate district; Nurse Corner, who comes from Leicester, has been appointed to fill the post.—A parcel received from Nurse Mary Trump, with many thanks.—We have also to acknowledge two shillings for the Leo Cot at the Children's Hospital, Cork, from Mrs. Goslett.—We hear that probationers will probably be received at the Mill Road Infirmary, Liverpool, early in the new year.

JOTTINGS FROM INDIA.—On October 25th a presentation of a valuable bracelet was made to Lady Roberts, as a farewell gift by her friends in Simla, and as a slight acknowledgment of her generous hospitality at Snowdon during Lord Roberts' long term of office. The Viceroy made the presentation in a very happy speech, the Commander-in-Chief expressing, on behalf of Lady Roberts, the pleasure and gratification with which the gift was received. A cheque for Rs. 1,300 for the Lady Roberts Fund accompanied the bracelet.—The outbreak of cholera at Murree, in which four officers and one lady died, has been attributed, among other reasons, to something they eat or drank at a ball there, as all who were ill had been present and were taken ill after it. A similar outbreak has taken place at Lucknow, supposed to be caused by the officers' cooking vessels not being clean, or the soup standing too long in one copper vessel. Captain Grierson, R.H.A., and the mess sergeant died of cholera on October 19th, and Captain Head, R.A., on the 20th. Lieutenant Sharp, who was also very seriously ill, has now recovered. Captain Head sat up all night with one of the others, and was ill only a few hours before he died. An inquiry is being made.

### The Royal British Nurses' Association.

The hearing of the application on behalf of the Royal British Nurses' Association for a Royal Charter was continued on Monday, the 28th ult., in the Council Chamber of the Privy Council at Downing Street. The Committee of the Council who heard the case were the Marquis of Ripon, Viscount Oxenbridge, Lord Hobhouse, and Lord Hannen.

The Royal British Nurses' Association was represented by Sir Horace Davey, Q.C., Mr. Muir Mackenzie, and Mr. W. H. Cross. Sir Richard Webster, Q.C., M.P., and Mr. L. S. Bristowe appeared for the opposition.

As on the previous occasion, the Chamber was crowded by ladies and gentlemen interested in the proceedings.

The arguments were completed when the Committee rose. It is not the practice in cases of this kind to deliver any judgments. Their Lordships merely advise her Majesty to grant or refuse the charter in accordance with the views which they have arrived at.

We do not venture to predict what the decision will be, but it will, we think, be obvious from a perusal of the report of the proceedings given below, that, even if a charter of incorporation be granted to the Association, there is little fear of their being enabled to establish any general register of the nursing profession, or to maintain any register of nurses at all which shall be compulsory, or shall even profess to be a guarantee of competence.

It may be mentioned that the charter sought to be obtained included a number of other objects, such as the founding and maintenance of schemes for the benefit of nurses in the practice of their profession, and in times of adversity, sickness, and old age, and the promotion of conferences and public meetings and so forth, besides registration. Counsel for the opposition stated explicitly, and most properly, that no objection was raised to anything except the registration scheme, and that whether or not a charter should be granted for the other objects, was solely a question for the consideration of her Majesty, with which the opposition had no concern.

The following report is *verbatim.*

The Marquis of Ripon: I am sorry, Sir Richard Webster, that I had to leave last Monday during your address, but I had to attend a meeting of the Cabinet. I have, however, carefully read all that you said upon that occasion.

Sir R. Webster's Speech.

Sir Richard Webster: I am much obliged to your Lordship. My Lords, on Monday last I had dealt with some general objections to the register and to the use that would be made of the register by the general undiscerning public. I now desire to make my observations somewhat more concrete, and to call your Lordships' attention to specific objections to this register, to specific objections to the society which proposes to manage the register, and to the powers that are sought to be obtained in connection with the register by the present proposed charter. Before I proceed to do that I wish to make one observation with reference to the general line of argument of my learned friend Sir Horace Davey. In answer to objections made by us, and to discount them —and properly so, from his point of view—he said: "All we ask is to make a Directory of Nurses." If all they really desire is to make a directory of nurses we should not be here to oppose this application, and further than that, I say, with great deference to your Lordships' better judgment and knowledge, that there is, as far as I know, no case and no precedent of a chartered register of any such body being directed. It is absolutely new, and it is open, as I submit, to the objections to which I will call attention later on. The charter cannot give the power which it is necessary should be given in order to make an effective register, but perhaps it is more important to put the case on its true ground, namely, that without statutory assistance such a register would be, and must be, misleading to the public, and would be dangerous. There is no precedent for giving such a register the sanction or *imprimatur* of its being a chartered register. My Lords, I now come to the objections to the register as it appears at the present time, and as it would appear under the charter. I will, first of all, ask your Lordships to look at the Association's register for 1892. That purports to be the register of trained nurses for 1892. It is not a mere list of members of the Association, but it is a register of trained nurses. I shall have to show your Lordships later on that one of two consequences must follow; either that every nurse desiring to have full opportunity of employment must join this Association, or else it will not, in any way, effect the purpose which the promoters of the chartered register desire. It was stated by my learned friend, I am sure by inadvertence, on instructions that the number of trained nurses was 2,777. That is a mistake; it is 1,777. But I am not going to deal with the mere question of numbers. The point is more important than that; 1,777 is the total number of nurses on the register out of about 5,000 trained nurses, and there are many more, variously estimated at between 5,000 and 15,000, who might, to a certain extent, and under certain rules, be allowed to come on the register. Your Lordships will kindly remember that the case made by my learned friend Sir Horace Davey was this: "We desire to enable the public to judge as to who are qualified and who are not qualified; we desire to guard the public against the danger of having unqualified nurses posing as qualified nurses, and passing themselves off on the public as competent to fulfil the duties of qualified nurses." I think your Lordships will be surprised when I tell you that upwards of 600 of these nurses have no hospital qualification at all; and I shall show, when I analyse this register, that so far from the nurses fulfilling the minimum qualification now put forward by Sir Horace Davey as being the main object and aim of this institution, a still larger number having nothing like the qualification which is supposed to be necessary in order that the public may not be misled.

The Marquis of Ripon: That is to say, the three years' qualification.

Sir Richard Webster: Yes, my Lord. I have already pointed out that, in the charter as it is now framed, there is no limit and no qualification imposed. Any nurse may be entered on the register subject to whatever regulations the Association may choose to make. I wish to call your Lordships' attention to the regulations as they are now in force, printed in the Register for 1892, at page 8: "Applicants for registration must produce proofs that they have been engaged for three years in work in hospitals or infirmaries, of which not less than twelve months must have been spent in a recognised general hospital containing at least forty beds." Then "for nurses working in a hospital the Registration Board may waive the production of other testimonials," and so on. I pointed out, when dealing with the constitution of the Association as defined by the proposed charter, that there was nothing to restrict or limit the qualification, and that it was open to the Association on the face of this charter to make any number of bye-laws. Lord Hobhouse observed to me that was true, but that still the charter might be modified, and restrictions might be imposed. But, my Lords, with submission, that will not get over the difficulty. How is it that out of 1,777 such a large number of unqualified persons were on the list up to June 30th, 1890, if the Association intended at that time that the register should have the effect of a medical register—namely, that persons, after a certain date, should not be allowed to join it without certain qualifications? That was their case when they went to the Board of Trade. Their case in effect was this: "We desire to be a certifying body, a body who shall lay down the minimum qualification for fit and competent nurses." Therefore, consistently with that, they were obliged to say— "Come and join us during the year of grace up to the 30th June, 1890, because after that you will not be able to join." So that the mode in which this register, which is to be put forward as the chartered register of trained nurses from year to year, has been formed, is by admitting before that period no less than 1,622 nurses. It is a little significant that out of 1,777 upon the register in 1892, no less than 1,622 joined during the period of absence of any qualification; and I may have to note presently that there has not been a corresponding increase since, for reasons which your Lordships may possibly more appreciate when the matter is fully expanded. Now, my Lords, in the first place, what is the qualification they themselves suggest? It is that the nurses should be engaged for three years in work in hospitals or infirmaries. There need be no hospital certificate; it is not necessary that they should have a certificate, but simply that they should show that they have been engaged for three years in work in hospitals or infirmaries. If your Lordships care to examine the Blue Book, I could show you, on the evidence of most experienced people, that in many cases a nurse with twelve months' experience is better than a nurse with three years' experience, that is to say, that there are varieties of nursing. It is a profession where an infinite variety of qualifications are required, as I pointed out on the last occasion, but it by no means follows that three years' training in a hospital is necessary or even desirable for many cases. I point out to your Lordships that if that were the test, and if that is supposed to be the minimum which the Council of the Association require, the state of things at present does not justify this register being sent out as a chartered register—a register which is to be kept under the authority of a Royal charter. This register of trained nurses would become, under Royal sanction, the Chartered Register of Trained Nurses for 1892. Now, my Lords, what is the position of things? Of the numbers that are now upon the register, 354 could be picked out at once as having no qualification at all. If your Lordships desired to examine into it I could give you the actual detailed cases, although I do not wish to mention names.

The Marquis of Ripon: No.

Sir Richard Webster: I can give your Lordships some specimens which can be explained by the register itself.

Lord Hobhouse: You had better give us one or two specimens by number.

Sir Richard Webster: There are 239 cases of no certificate, and no evidence of competency at all. [The learned counsel then referred to several nurses whose names appear in the Register. He pointed out that the first was stated to have been at "Guy's Hospital, 1872-73," and continued:—] The period mentioned is 1872-73. That cannot be three years, and it may, of course, have been only for a short period of a few months. The other institutions mentioned after that name are simply nursing institutions—most estimable places, of course—from which nurses are sent out. Then I will take the next one. That is Tottenham Hospital. I should tell your Lordships that Tottenham Hospital is a Deaconesses' Home, and does not confer anything like the same qualifications as a general hospital. There is no reference to the time, or a statement of the time, which the lady has passed in that place. I mention these instances, not to depreciate particular individuals the least in the world, but simply to show that, spoken of as a register of qualifications having the *imprimatur* of the Association, it becomes really anything but a safe guide.

The Marquis of Ripon: I should like to ask you for an explanation on one point. It is stated with regard to those marked with a dagger, "These statements have not been verified by the return of the printed circular."

Sir Richard Webster: They purport to send out, my Lords, every year a circular. I am told that it is a circular simply asking whether the name and address are correct. That note means that they have received no answers from those who are marked with a dagger, and no less than 312 out of the 1,777 are marked with a dagger, which means that they have not been verified up to date. I mention that because it bears on another important point which is not disputed in fact, but the weight of which I think has not yet been quite appreciated, namely, the impossibility of keeping such a document as this by any general body. Then the next is the "Leeds Infirmary, 1880." It is obvious that that nurse could not have been any length of time in the hospital. She must have been there less than a year. [The learned counsel then referred to another name stated to have been at the Miller Memorial Hospital, Greenwich, and proceeded:—] That is a small memorial hospital where there are only 23 beds, and the statement is, as regards that hospital, "in 1885." That will indicate to your Lordships at once, if you deal with the register from the point of view of information, that that cannot be the qualification of a trained nurse, or even approach the qualification of a trained nurse, which they suggest is the minimum they are willing to put on the register.

Lord Hannen: Are not these cases covered by the note that the Registration Board has power to waive this latter condition?

Sir Richard Webster: No, my Lord, not one of them. On page 8 your Lordships will see this: "Applications for registration must produce proof that they have been engaged for three years in work in hospitals or infirmaries, of which not less than twelve months must have been spent in a recognised general hospital, containing at least forty beds." What may be waived is the latter condition as to the number of beds, but the condition of three years is supposed not to be waived under any circumstances. I have now, my Lords, handed to me the circular to which Lord Ripon was good enough to call my attention. It is in these words: "Madam, will you kindly correct, if erroneous, the accompanying slip from the register, and in any case send the same by return of post to the Registrar. Statements not verified or corrected by the nurse will be indicated by a sign." From the point of view of ascertaining whether the nurse was a fit and proper person to continue a trained nurse, that would be simply nugatory. No nurse could be expected on such a slip to disclose anything that would interfere with her own competence. It only amounts to this—is your name the same, and is your address the same as before? Your Lordships observe that the qualifications are only those that had been previously investigated.

Lord Oxenbridge: The case to which you referred last is a little peculiar. Some of them have such and such a year, and then it goes on "to date." But there the date is omitted. It is simply "Miller Memorial in 1885."

Sir Richard Webster: I do not suggest, of course, that my criticisms are exhaustive, but I have been able to find, by my instructions, a very large portion of cases that do not come anything like within the qualification that they suggest should be the minimum.

The Marquis of Ripon: That is the case of a nurse engaged in private nursing.

Sir Richard Webster: Yes, my Lord. Then I may refer to another, whose qualification is stated to be "Henrietta Street Institution, 1867, to date; St. Bartholomew's Hospital, and the City of London Lying-in Hospital." No dates are given, and therefore it is a little difficult to trace those. But in that case there are no hospital qualifications of any sort or kind. The only statement is that at some time or other the nurse has been at St. Bartholomew's Hospital and the City of London Lying-in Hospital. In this way, with reference to the information contained in the register as to the qualification of the nurses (and I do not wish to weary your Lordships by simply going through numbers), I can show that 354 out of the 1,777 upon the register are nurses who will appear on the register of trained nurses from year to year, with the statement that the Association themselves are from time to time revising the list, but yet they have not got anything like the minimum qualification which is suggested. Then, my Lords, there is another matter which certainly requires serious consideration. If your Lordships will look at our printed case, page 86, it will be seen that there may be discovered 25 cases of mistakes in the list. The names are not given, but the name and numbers can be given if necessary. The qualification given is: "London Hospital, 1884-85." That is a case where the name has been traced, and in that instance the nurse was only there seven weeks on trial. Then, "London Hospital, 1883-84," the nurse was only there three months. And "London Hospital, 1879-80;" again only a period of three months. Therefore, your Lordships will observe that the statement made of the hospital training which is all that the register requires, is not, in the first place, a statement which gives the length of time, and is distinctly misleading to the public. Further than that, more serious errors have been discovered. There are on this list the names of nurses who have been dismissed from the hospital for misconduct. It can be proved before your Lordships if necessary (and I do not know whether the opportunity will be afforded or not) that with regard to the compilation of this register in the case of a large number of those who appear as connected with particular hospitals, no application of any sort or kind has been made to the hospitals with reference to the individuals, although in a number of cases the hospitals have had private and confidential information.

Sir Horace Davey: My Lords, I am instructed that that is quite contrary to the fact. Information has been refused.

Sir Richard Webster: Of course, my learned friend and I speak from instructions. My instructions are (and I am prepared to put evidence before your Lordships on the subject) that both as regards the London Hospital and St. Thomas's Hospital no application of any kind has been made with reference to the large number of nurses whose names appear as having been qualified at this hospital, although in many cases private information has been at the disposal of the hospital authorities. The hospital authorities, when they are asked for a nurse, could only send her out in accordance with their own information as to her own fitness.

The Marquis of Ripon: As I understand, you say that no application has been made to those hospitals in any case.

Sir Richard Webster: No, my Lord, I did not mean those two hospitals alone. There, again, I am prepared to prove it. I am informed, speaking of the large number of the hospitals for which I appear, seven altogether which have training schools, no such application was made to any of the hospitals prior to the putting of the name upon the register.

The Marquis of Ripon: In the first instance.

Sir Richard Webster: In the first instance, my Lord, and in the great majority of hospitals no application has been made to them at all with regard to the fitness of the nurse. There have been quite lately applications made with reference to some particular nurse as to which information was sought and given.

Sir Horace Davey: Refused.

Sir Richard Webster: With regard to the application made before the names are put on the register, or with reference to the annual continuance of the name on the register, my instructions are no application of any kind has been made either to the hospital or to the training schools. This is not a case which depends upon the statements of counsel merely, because, if necessary, and your Lordships required it, it can be proved, and I am in a position to tender evidence, if your Lordships think fit to receive it, with regard to this matter. But it is essential before we consider the question of evidence that I should open this case fully to your Lordships. Would your Lordships kindly look at another case.

The Marquis of Ripon: I do not know that we want any more of these individual cases. You have given us the general nature.

Sir Richard Webster: Of course these have been given to me on the authority of gentlemen from the training schools.

The Marquis of Ripon: If you have any singular case particularly striking you wish to bring forward do so, but I do not think we need further ones.

Sir Richard Webster: I do not want to bring them forward; it is not pleasant.

The Marquis of Ripon: You have illustrated your point.

Sir Richard Webster: Cases of nurses really dismissed for misconduct, and not found fit to be trained, and who left the hospitals for that reason, still appear on the register. I do not bring them forward with a view of saying one word against the Association if it is confined to its proper work, but it is essential, and it is a necessary consequence of a public body of this character attempting to do work it is not qualified to perform, that these errors must creep in, and your Lordships will have noticed—because your Lordships heard my learned friend Sir Horace Davey's speech on behalf of the petitioner—that they do not deny the extreme difficulty of getting the names of incompetent nurses off the register. They admit they would not get legal testimony, and that persons having the legal evidence would not put it forward. The statements in the register would not be privileged; the conduct of the managers of the Association would not be privileged; and later I shall have to show your Lordships the distinction between this and the case of the medical register, and show your Lordships what are the safeguards Parliament has thought fit to enjoin in connection with registers of this class.

The Marquis of Ripon: I am right in supposing that the charter would not cause these statements to be privileged?

Sir Richard Webster: No, it would not in any way, nor would it be possible to protect the persons by charter; nor would it be possible to impose penalties to prevent false statements, nor would it be possible to make it an offence to improperly represent yourself as on the register of trained nurses. That is a branch of my argument to which I shall ask your Lordships' serious attention presently, but your Lordships understand it did not enter directly into the matter I am discussing at the present moment. My Lords, I need not say that to my learned friend or his advisers any information that we have—because the matter has been most carefully gone into with reference to the particular cases—is at their disposal as much as mine.

Sir Horace Davey: It has been refused.

Sir Richard Webster: My learned friend must pardon me. It is better we should conduct the case with the ordinary recognition of courtesy one to another. There has been no refusal of the matter I am offering to my learned friend at all, because it has been done for the purposes of this case. The register has been examined, and I am in a position to give my learned friend the same materials that I have, namely, as to the insufficiency of the register, and the sum total of it is that of the 1,777 cases there are 354 cases which under no circumstances ought to have been put on the register of trained nurses, having not the proper qualification for that designation. Those appear on the face of the register, except in some eight or ten cases from various hospitals where information has been received as to the special causes which might not be discovered, and could not be discovered by the framers of the register. Now, my Lords, I come,—passing from that, though it is certainly a most important point—to what I call, if I may give it that name, the change of argument, by which this case has been supported. As I said to your Lordships but a few moments ago, over and over again my learned friend said, "We do not aspire to any of these things; we do not want to be regarded as an authentic register; we do not desire to be more than a Directory of Nurses." I say again, parenthetically, I know of no such thing as a chartered directory of anybody. I do know a medical register under a Statute, and I do know a register of veterinary surgeons under a Statute, and a register of pharmaceutical chemists under a Statute, but I have done my best to obtain information from your Lordships' officials, and I am informed that this application is without precedent.

Lord Hannen: One name occurs to me—I daresay you will point out the distinction—Chartered Accountants.

Sir Richard Webster: The Chartered Accountants have no power to keep a register of any kind. All they have power to do is to allow members to join their association, and we have not the smallest objection to this association keeping a list of its members, and if your Lordships think right, if a case is made for it, even by Royal charter to get their members to join; but what those gentlemen who instruct me feel so strongly—and I trust I may be permitted as an advocate only to endorse what they say—is that the evils consequent upon a chartered register, of itself an entirely novel thing, in the case of the profession of a nurse, are a hundred-fold what they would be in the case of anybody else, because of those matters to which I have referred, at perhaps too great length, last Monday, namely, it not being possible to produce the essential qualifications of a nurse on the register.

Lord Hannen: Would it be illegal for the Chartered Accountants to have a register?

Sir Richard Webster: Not at all—a register of members. That is not what my friend wants.

Lord Hannen: That is all that they would get. I am not disregarding your argument that it would be misleading. That is another branch of the subject.

Sir Richard Webster: Would your Lordship look at page 4 of their charter? "The purposes of the Corporation are the following: 1. The maintenance of a list or register of nurses showing as to each nurse registered her name and address, and the name of the hospitals or other places at which she has been trained." I have never suggested that it would be illegal to keep a list of members or publish it, but I know of no case—and if your Lordship thinks there be a case, your Lordship will let me deal with it, I am sure—in which the purpose of the chartered corporation has been to keep a chartered list, if I may put it, in a compendious way. Your Lordship's observation has led me to make the point now. If they only want to make a list of their members, they can do it by their bye-laws. There is nothing in the world to prevent their publishing a list of their members, but why they have been desirous of obtaining this chartered power is to make the register the authentic and recognized organ in which the names of trained persons are to appear, and they do not hesitate to say that their ultimate object is to prevent people not upon this register from being considered to be properly qualified trained nurses. It was not until we were before your Lordship's Board, and until my friend Sir Horace Davey opened the case, that there was a suggestion that all they wanted was to make a directory of nurses. That, of course, is a strong observation, and must not be taken from me unless I can make it good. I will call your Lordship's attention in the first place to the evidence, because that is on this point very important. I will show your Lordships that the object of the Association has been a general register, and that that object has been maintained down to the time of this petition, and is, practically speaking, endorsed by the petition itself. At page 553 of the first Report of the Committee of the House of Lords on Metropolitan Hospitals, the lady who has done more to promote this Association than anybody, Mrs. Bedford Fenwick, is giving evidence—a lady of immense ability and energy and powers of organisation—and at question 9,604 she is asked, "As to this British Nurses' Association"—that is, the applicants before your Lordships—"are you connected with that? *A.* Yes. (9,605) Will you tell us what it is? *A.* It is, firstly, to unite trained nurses together in a purely professional union; secondly, to provide for the legal registration of nurses under the control of medical men; thirdly, to help nurses in times of need or adversity; and, fourthly, to improve the knowledge and usefulness of nurses throughout the empire. (9,606) Do you consider it is necessary to have such an association as that? *A.* I think so, under the present circumstances. Nursing has so improved—I may say so advanced—in the last ten years, that I think now we have now come into a condition in which, as a profession, it should be organised; and I believe that the only way of organisation of the profession is by giving the members of that profession a voice in their own progress and education, and also by having a controlling body outside the general committees of our individual hospitals, who would be in a position, I may say, to regulate the education and also the condition of the nurses. It is really a matter practically very much the same as the medical profession. Although all our medical schools in London have their own basis of medical teaching, still there is a General Medical Council, which controls the whole profession, and regulates, I may say, the education of medical men; and I believe that it would be a very good thing if nurses could be, to a great extent, controlled in the same manner, because although hospitals have entire control over nurses as long as they are in their service, when they leave the hospital they are now entirely irresponsible persons; they can do what they like. (9,607) That is to say they have got their certificate? *A.* They have got their certificate, and hospitals cannot recall their certificates. Nurses may be accused of grave faults; they may be accused of crimes and be imprisoned, but when they come out of prison they are quite at liberty, if they choose, to attempt to get, and do get, work again upon the score of their certificate. (9,608) Then you think it is necessary, in order to protect the public, that there should be such an Association? *A.* I think first, for the protection of the public, that there should be a control over all trained nurses. Untrained or semi-trained nurses are under the control of their individual committees. (9,609) Have you one grade of certificate or various grades? *A.* With regard to our Association we do not give a certificate, but we give those nurses who are registered, I suppose you would call it, a certificate of registration. The certificates given by their various nursing schools are registered. (9,610) But then, under that plan, what greater protection is there for the public than comes from the hospital? *A.* No hospital is responsible for a nurse once she has left the hospital service; but a General Nursing Council or Registration Board would be responsible to the general body of nurses and to the public to prevent any woman who proved herself unworthy of trust, going on with the work. They would take her name off the register." Now, my Lords, that is not a Directory of Nurses. That is a certificated register of nurses which these ladies want it to be; they want it to be the register of nurses under the *imprimatur* of the Royal charter. Then question 9,611, "You would keep in touch with each nurse, you mean? *A.* Yes; just as a medical man can be struck off the register, a qualified medical man, for gross professional faults or crimes, so a nurse should be. It would not be right to give any one individual hospital committee the right to do so. The register would be published like the 'Medical Register,' the 'Law List,' the 'Clergy List,' and similar volumes every year, and be on public sale; so that anyone could, at a glance, see whether any given woman was a trained nurse or not, and if so, what exact training she had received." I may say parenthetically perhaps that some of us know more about the 'Law List' than Mrs. Bedford Fenwick. I doubt whether the 'Law List' or the 'Clergy List' would give quite the same kind of information. Then passing by the reference to the Pension Fund which covers another point, I would call attention to question 9,623, at page 554: "Then if you had a charter or an Act of Parliament you would have this much larger power, which would enable you to strike off nurses from your list, and you would have considerable powers of enforcing discipline? *A.* Certainly. *Q.* (9,624) [Earl of Kimberley]: You can strike them off your list? *A.* Yes; but that would not have the same moral influence. (9,625) [Earl Cathcart]: Do you agree with Dr. Bedford Fenwick"—Mrs. Bedford Fenwick is Dr. Bedford Fenwick's wife—"that those powers would be desirable? *A.* I believe so. (9,626) Namely, by Act of Parliament or by charter? *A.* Yes. (9,627) And to do what?"—will your Lordships note this—"By granting a Royal charter to the Nurses' Association to confer legal powers of control and discipline over the registered nurses, or by a short Act of Parliament, appointing a Registration Board, and ordaining that no public or private institution should send out women to nurse the sick who were not duly registered. To my mind the latter would be the better way, by far."

The Marquis of Ripon: That is by Act of Parliament?

Sir Richard Webster: Yes. But the former could be brought into operation at once, and would give a strong basis for improvement and future Parliamentary action.

Lord Hobhouse: There seems to be a great confusion here between Royal charters and Acts of Parliament.

Sir Richard Webster: Certainly.

Lord Hobhouse: I do not know what powers the charter could give.

Sir Richard Webster: And the gentleman who drafted this charter certainly had the most extraordinary ideas of the powers of these charters. I am only at the present moment endeavouring to point out that the kind of list proposed to be obtained by the present charter is obviously the last resort of clever men, to whom what the Association really wanted to get did not commend itself. Would your Lordships kindly look at page 7 of their draft charter, under the head of bye-laws: "At any general meeting it shall be lawful for the members of the corporation, or such of them as shall be then present, to ordain and make such bye-laws as to them, or the major part of them, shall seem proper for the regulation and good government of the corporation and of the members and affairs thereof, and generally for carrying the objects for which the corporation is founded into full and complete effect, with reasonable penalties and fines to be contained in such bye-laws on the offenders for non-performance of, or disobedience to the same and the said bye-laws, penalties and fines, or any of them, from time to time to alter, change, or annul, as the said members in general meeting shall think requisite, and to mitigate the same as they shall find cause, so as all and singular such bye-laws, penalties, and fines be reasonable, and not repugnant, or contrary to the provisions of these presents or to the laws and statutes of this our realm." My learned friend Sir Horace Davey's experience of these matters is ten times more than mine, but I know of no power to impose fines by charter. I point out that they recognise that unless there is a machinery which will make this an effective register the Royal charter is not wanted.

Lord Hobhouse: I asked Sir Horace Davey what powers could be gained by a charter which would not be gained under the Joint Stock Acts.

Sir Richard Webster: Not so many.

Lord Hobhouse: I understand him that nothing but the greater permanence of the institution.

Sir Richard Webster: Not so many powers. My friend, Sir Horace, was perfectly right. I shall say to your Lordship later on that if it were a question of obtaining incorporation and powers, the incorporation under the Limited Liability Acts would do more than what they get by charter.

The Marquis of Ripon: Their contention on that point is that they have been refused by the Board of Trade to have those powers without taking the word "limited."

Sir Richard Webster: My Lord, I am coming to that when I deal with the Board of Trade incident, and I shall point out that the objection to that grant was on the ground that what they proposed to do would not effect its object—not that it would not be a good thing to obtain incorporation, but that they would not obtain their object by it. As I have referred to it I can go on with that point.

The Marquis of Ripon: Never mind; I just interjected that remark.

Sir Richard Webster: Why should not I give it to your Lordship now. It is at page 71 of our case. The letter of the Board of Trade is dated May 6th, 1891, and it says—"I am directed by the Board of Trade to say that they have carefully considered the application for a licence under Section 23 of the

Companies Act, 1867, authorising the Royal British Nurses' Association to register as a limited company without the use of the word 'limited.' The Board of Trade have received a large number of communications from bodies of persons whose interest in hospital nursing is unquestionable, and whose experience entitles them to speak with authority, strongly objecting to the issue of a license. After careful consideration of the objects of the Royal British Nurses' Association, and of the representations made in opposition thereto, the Board of Trade are unable to satisfy themselves that the means which the Association propose to adopt are either adequate to carry out their objects satisfactorily, or so free from objection as to warrant the Board of Trade in the issue of a license, and, under those circumstances, they are unable to accede to the application. I am, however, to point out that this refusal in no way precludes the Association from registration as an ordinary Joint Stock Company, under which registration they would enjoy the same powers and be subject to no greater responsibilities than would be the case if they were registered without the word 'limited.'" I should not read this, perhaps, except that my learned friend read a letter, of which we have never seen a copy, suggesting that the Board of Trade were supporting the charter. Perhaps I may see that letter now. According to my instructions we had never seen that letter till it was read, but I should like to see the whole of it, because I do not know under what circumstances it was written. Under no circumstances can it be said to be a withdrawal of the reasons given in Sir Henry Calcraft's letter. Then if your Lordships look at page 72 of our case, you will see that Sir Michael Hicks Beach was asked by Mr. Paulton in the House of Commons "whether the application of the Royal British Nurses' Association for permission to omit the word 'limited,' if registered under the Companies Act, had been refused by the Board of Trade?" In answer to that Sir Michael Hicks Beach said: "After careful consideration of the application to which the hon. member refers, and also of the representations made to me in opposition to it by bodies and persons whose interest in hospital nursing is unquestionable, and whose experience entitles them to speak with authority, I was unable to satisfy myself that the means which the Association proposed to adopt were either adequate to carry out its object satisfactorily, or so free from objection as to warrant me in complying with the application made to the Board of Trade." Therefore, I point out that it is not to get powers; they can get the powers to-morrow—they can get the incorporation; but they must call themselves "Limited" for this purpose. It is obvious, as we humbly submit, that under colour and with the sanction of a Royal charter, this register will have a different effect to that which it would have if it were a mere list of members, and the distinction does appear to us, and we humbly submit it would so appear to your Lordships, on consideration, to be one of great importance.

The Marquis of Ripon: You mean a different moral effect?

Sir Richard Webster: And practical effect.

The Marquis of Ripon: Not a different legal effect?

Sir Richard Webster: Not a different legal effect, but a different practical effect—a different operative effect to the people to whom it would speak. And this Association themselves say that unless people are on this register they shall not be considered trained nurses at all. While the letter which I have asked for is being looked for, will your Lordships look at the answer which is given on page 555 in the same volume of the Report of the Lords' Committee. I need not read the question which has a poetic quotation in it. "I do not think that we desire to register personal qualifications of that sort. What we want to do is to place the skilled nurse on safe ground—that is, that when she has gone through a certain training and knows a certain amount, she should not have to compete in the open market with unskilled nurses; and that the public should be protected from any amateur and bogus nurse who may don a cap and apron with very little training, and take the same amount of fees from the public as are paid for a trained nurse." Having regard to what I have shown your Lordships with regard to their own register, a somewhat startling criticism is to be gathered from Mrs. Bedford Fenwick's own answer; but I ask how are the public to be protected. It is only by the register. No other means are suggested which will protect the public, and, therefore, it is obvious that the protection is to be by certain names being upon the register, and certain names not being there. Therefore, again, I say with great deference, to call that a directory is simply to put your head in the sand and fail to observe the difficulty which is connected with the particular matter in dispute.

Mr. Muir Mackenzie: Here is the letter. It is a very long letter indeed.

Sir Richard Webster: It had better all be read. I see this letter is dated August 6th, 1891. Sir Henry Calcraft's letter was on May 6th, and Sir Michael Hicks Beach's answer was June 19th. It is Sir Henry Calcraft to Dr. Bedford Fenwick: "With reference to your letter of the 31st ultimo, and to previous correspondence, with regard to the application of the Royal British Nurses' Association for a licence, under Section 23 of the Companies' Act, 1867, I am directed by the Board of Trade to state as follows: The Board do not propose to enter into a discussion with you as to their legal powers under the section in question, as they are unable to see that any useful purpose would be served by so doing. If the Royal British Nurses' Association is of opinion that it can legally contest the decision at which the department (in the exercise of their statutory discretion) have arrived in the matter, it is of course open to it to do so in such a manner as it may be advised. With regard to your statement that the decision of the Board of Trade, not to grant a licence in this instance, was announced without affording the Royal British Nurses' Association 'any opportunity of stating its case,' I am to observe that the decision of the Board was arrived at upon the facts and circumstances actually before them. If the case of the Association was inadequately stated when the original application for a licence was made on its behalf, that is not a matter for which the department are responsible, and, however, this may be, the Association has since had every possible facility afforded it for re-stating its case after a perusal and consideration of all the documents before the Board of Trade in the matter. You have now availed yourself of these facilities and have re-stated the case for the Association. After commenting upon the alleged uninfluential and untrustworthy nature of some of the objections sent to the department, you state that some of the persons who made them were unaware of the facts, and you add: 'In the most important petition of all to which well-known medical men have complaisantly added their signatures, nearly every statement is utterly inaccurate.' On these points the Board of Trade are unable to accept your statements as conclusive. The fact remains that (in consequence of adopting their usual action in requiring the application of the Royal British Nurses' Association to be advertised), the Board have been made aware of a strong opposition to it on the part of the authorities whose position renders their opinions of great weight on such a matter. It appears to the Board of Trade that they are not competent to determine the very important questions connected with the establishment of a register of nurses which should be settled before the register can be effectively established. Some of these questions are of great weight. Under these circumstances the Board of Trade are unable to grant the desired licence to the Royal British Nurses' Association. They wish it to be distinctly understood that they are led to this decision by no hostile feeling towards the Association or its objects, but by a conviction that full inquiry (by competent authorities) into all the facts and circumstances of the case, and into the objections that have been raised, should precede any further steps on the part of Her Majesty's Government. As evidence of the position taken up by the department in the matter, I am to state in conclusion, that, should the Association decide to make an application to the Privy Council for an inquiry with a view to obtaining the powers they desire, such application would receive the cordial support of the President of the Board of Trade." Now, my Lords, the whole point turns on an application to the Privy Council for the powers that they desire and the powers that they were then desiring were powers, as I shall show your Lordships in a moment, to make this register a useful record of competent nurses, instead of a misleading record as it must be under the circumstances to which I have been calling attention.

Lord Hobhouse: You mean all the Board of Trade cordially support is an inquiry?

Sir Richard Webster: Yes. Perhaps I should say now, my contention is that such powers which are sought directly or indirectly ought only to be given after a full Parliamentary inquiry, where the merits of the case can be discussed and evidence taken, and that your Lordships ought not to be asked to prejudge without inquiry, for really

I never heard of evidence being called before the Privy Council on such an application; there may have been, but I do not know of it. I say your Lordships ought not to be asked to express an opinion in favour of a chartered register of nurses if your Lordships think there is even ground for inquiry into the allegations we have made, and which, my Lords, as I shall show later on, are supported by nine-tenths of the people who have been engaged in training nurses for thirty or forty years. Then, my Lords, returning to the evidence before the Lords' Committee. At page 566 of the first Report Lord Kimberley puts this to Mrs. Bedford Fenwick, at question 9,667: "About the legal powers which you spoke of, will you tell us a little more precisely what they would be? *A.* A Royal charter or a short Act of Parliament would probably appoint a registration board, composed of the leaders of the medical profession, who are interested in nursing, and of hospital matrons with great experience. It would empower them to overlook the certificates which nurses could bring forward, and to register them upon these certificates. Of course, they would have to rely upon the certificates which they received from their training schools, so that the matter still remains to a great extent in the hands of the authorities of the training schools. If they did not choose to certificate a person as an efficient nurse she could not be registered." 9,638: "I thought you intended it to become a prohibitory power, to prevent nurses not belonging to the society from being sent out to nurse. I may have misunderstood you?" —"I think it would act directly upon that matter, because the public would soon recognize the fact that when they paid a good fee for a nurse they wanted a trained nurse, and not a quack, just as when they go to a medical man, instead of going to a quack, it is of their own will, not that they are obliged to do it."— How would the public recognise it except by their names being on the register—"I wanted to know whether your Association would seek merely for power to deal with the nurses who might place themselves of their own accord upon your register, and with regard to whom it would be right that you should have power to deal with them yourselves, or whether you would wish to take any power to prevent nurses from performing any nursing duties without your sanction. *A.* I believe that if a Royal charter or an Act of Parliament is granted that would be the effect, that no nurse would be considered a trained nurse if she was not registered." Now I am justified in taking the belief of Mrs. Bedford Fenwick, who is the main promoter of this application, and who has done more to work up the agitation than anybody. I am justified in taking that in pointing out to your Lordships that her view at that time—in July, 1890—was that the result would be that no unregistered nurse would be considered a trained nurse. My Lords, may I for a moment break off to point out to your Lordships that the qualification they suggest is of itself not a good one, because they have admitted on their register a large number of persons who should not be on it, and that it was with the object of admitting them that they fixed a certain day of grace, after which they would not be let in? Does not what I have stated show that every step they have taken is to do something to make the register speak to the public in the way in which Mrs. Bedford Fenwick wished it to speak, so that no nurse would be considered a trained nurse if she was not registered? Assume that your Lordships were satisfied that in many cases of private nursing a year in a hospital was better than three—assuming a lady was competent, and showed great skill in nursing, that taste and that lightness of touch which I believe is absolutely essential for being a thoroughly good nurse—assuming she shows that and qualifies in a year, the Association says: "No; we will have three years in a hospital." That lady, perhaps as competent a nurse as can be suggested, is excluded from the register, with the result that the effect of the Royal charter, if granted, would be that no nurse would be considered a trained nurse if not registered. Then the evidence continues: "Your proposal is, in fact, to establish a close corporation? —In the same way only as the medical profession is a close corporation (9,641). Have you ever considered what the result of that would be if it were applied throughout the country; would it not be likely to produce the most extraordinary embarrassment and difficulty?—I do not think so (9,642). Do you think there are likely to be a sufficient number of trained and certificated nurses provided to serve patients throughout the country?—With all the enormous amount of training going on now in our large hospitals, which are turning out more nurses than can get work, I think it is hard that they should have to compete in the open market with so many amateurs." My Lords, I am informed that if what my learned friend said should be the minimum of qualification (and he was instructed to tell your Lordships that the opposition only came from people who desired to have a less qualification than the Association wished to put forward—certainly not quite, I think, proper instructions, having regard to what my clients have said) were to be the qualification it would reduce the present number of nurses to one-third, or, in other words, that there are left competent nurses who have been carrying on their profession for years who have not joined the Association, and who could not join the Association if that was the minimum, and who would be excluded from the category of trained nurses, and *ex hypothesi* on the present statement, would come to be regarded by the public as not being qualified to call themselves trained nurses.

Lord Oxenbridge: You see in the reply of Mrs. Bedford Fenwick to the Earl of Kimberley, on page 551, question 9,557, it is put very strongly.

Sir Richard Webster: "How long do you think a nurse ought to have been trained in a hospital before she is sent out as a private nurse, as a trained nurse?—I think she ought not to be sent out to nurse the sick until she has gained a certificate, which is the guarantee of the hospital which trained her that she is an efficient nurse. In most of the large hospitals I think that three years is the time which is required, and I think that is quite the minimum time for which she should be trained before she is sent out."

Lord Oxenbridge: That is in connection with what you said about the minimum time.

Sir Richard Webster: I point out that there are a body of persons, Miss Nightingale amongst them, who have come to the distinct conclusion that speaking of trained nurses for private nursing, assuming there is aptitude, assuming there is talent, assuming there is disposition and moral character, a year is ample to learn the technical work, and having regard to the hardening influences, rendering persons to a certain extent callous, as must, I fear, in some cases happen when they have to see repeated suffering, the longer training in the hospital has not, in some cases, an improving tendency.

Lord Hannen: If the public take that view of it they would not look to this register.

Sir Richard Webster: You are perfectly right—if they did.

Lord Hannen: Why should not the public have a guide to nurses who have served three years?

Sir Richard Webster: Certainly, if that is all that is sought, but that is not what is put forward.

Lord Hannen: The rest is speculation and anticipation.

Sir Richard Webster: Your Lordships will pardon me for a moment. That really is not so. This has been published, and it is the "Register of Trained Nurses for 1892" —not the members of the Association.

Lord Hannen: Assuming that to be a good objection, that would be remedied by simply inserting, "Register of Nurses," and then giving the title.

Sir Richard Webster: But your Lordships will pardon me for a moment. That is not quite, if I may be permitted to say so, the position in which the opponents are placed.

Lord Hannen: But that is what we are considering.

Sir Richard Webster: I quite agree, but I have come here to contest an application for a charter with a certain definite object, and that was not to simply prepare a list of their members; and, as I have said, that even does not meet another main objection, namely, that it is to be one of the purposes for which the chartered company or the corporation are to be incorporated. A list of members is only a matter of bye-law; a list of members is not a matter for the objects of the incorporation. The object of the corporation should be to improve the members, to promote a certain art, to promote a certain study. If the object be to keep a chartered register, it must mean that your Lordships think that it is a desirable thing that the proper function, so to speak, of the chartered corporation should be to keep the chartered register. Now, my Lords, would your Lordships kindly look at page 557 of the first Report of the Lords' Committee, question 9,649? Lord Kimberley puts this to Mrs. Bedford Fenwick: "And you further pointed out that if the hospitals recognised the utility of such an Association, they would require their nurses probably to register themselves? The moral force of the thing would compel all the trained nurses to become registered in the course of a few years; still that would not prevent the amateur nurses still being in existence any more than the quack doctors." If your Lordship (Lord Hannen) will bear with me for a moment, if I refer to your Lordship's own observation, I am not for a moment suggesting that something may not be put into this charter which would guard against the evils which my clients feel so strongly, but, on the other hand, if we had not brought this to your Lordships' attention and brought to your Lordships' notice the object and aim of the persons who are promoting this, and the unveiled desire that this register should become the official list of nurses, your Lordships would have had a very just reason to complain that you had granted the charter under a misapprehension. If, after considering what we have put before your Lordships, your Lordships think that evils do not exist, and think there is no room for Parliamentary inquiry, or that no Parliamentary sanction is necessary for such purposes as are indicated in this charter, of course our duty will be performed, but at any rate I think your Lordships will not think we are wrong in bringing this to your Lordships' attention, because a register is, in the eyes of the promoters themselves, the important consequence and the important object with which the Association is worked.

Lord Hannen: I am only pointing out that a very slight alteration would meet that objection. It would be simply to transpose the lines and read it thus: "The Register of Trained Nurses for the year 1892 of the Royal British Nurses' Association." Those words are all there. It is only a little different order.

Sir Richard Webster: The real vice is in the words "trained nurses." It is in that expression lurks the danger. It is the Register of Trained Nurses. It is an attempt to get a special sanction to that word "trained." If it were "The Register of the British Nurses' Association for the year 1892," it would be absolutely free from objection. But it would not effect their object. They would not be here for the charter if that was all they desired. Now, my Lords, I desire to show your Lordships that this is what they have been up to the present time seeking, and I will ask your Lordships to be good enough to refer to the documents that are in our case. I have several documents, but I will only refer to two or three. If your Lordships would look at pages 52 and 53 of our case there is an extract from the first annual report of the Association. It says that the General Council authorised the following schemes to be undertaken. The first is, "The formation of registers of nurses and of midwives"—not a list of members, but a register of nurses. Then it proceeds:—"In fulfilment of this programme and of the primary purpose of the Association, the petition for a Royal Charter, the General Council has to report that the subject of registration has been most carefully and thoroughly considered. It is clearly recognized that it is a great scheme for an Association to undertake. But in view of the undoubted evils which are extant in our midst, of the impunity with which any woman, however ignorant of nursing and midwifery, can term herself a trained nurse or midwife, and obtain employment, and bring about great danger to the sick by pretending to act in either capacity; of the powerlessness of the public or of medical men to prevent such women so working, or even control certificated midwives and trained nurses who become unworthy of trust; and of the material and moral injury to well-trained nurses and midwives, and to the good name of the nursing craft, which such women are daily causing, there can be no reasonable doubt that some remedy is urgently demanded. The British Nurses' Association suggests registration of trained nurses and midwives as a system which has worked excellently well in protecting the public from impostors in other skilled callings. It has endeavoured to persuade State authorities to undertake the scheme, but one and all have declined to commence so great a work. The Association, therefore, must firmly determine to initiate registration itself. It is supported in this by many of the leaders of the medical profession. It is opposed by some who make vague assertions of evil, but will give no definite facts in proof of their statements nor any advice or assistance to the Association in formulating any other remedy, although they do not attempt to deny that the grave evils before mentioned are in existence and on the increase. The General Council, therefore, having asked for this advice and assistance from every one who can give it, finds itself obliged to act on its own initiative. It has decided to open, without further delay, the registers of nurses and midwives, and to place on these at once, without payment, the names of members of the Association"—so that the members of the Association would go on at once—"For a few months to come all who have been engaged in nursing or midwifery for three years, and possess unexceptionable testimonials of knowledge and character, will be eligible for registration. But after this brief period of grace and thenceforward only those women who produce certificates of training from a recognised nursing school, or diplomas from an obstetrical examining body, will be entered on the registers. When a sufficient number has been enrolled the General Council proposes to present the petition for a Royal Charter to her Majesty the Queen in Council." Therefore I point out, with great deference to your Lordships, that it is not a question of simply having a list of their own members. It is not a question of publishing the names of those who join; it is not a question of gaining strength, weight, and popularity by *bonâ fide* advertisement, but it is by alleging to the world that they are the only chartered Association that is allowed to issue a register of trained nurses, because there can not be what I may call a succession of chartered registers. In that connection I shall have to say something later on, but I am dealing with a matter of principle now. Then, I think, I can take your Lordships at once to page 60 of our case, which contains the memorandum presented by the Association to the General Medical Council in 1889. Paragraph 5 says: "The cardinal principle for which the British Nurses' Association has striven, in the face of enormous opposition, is that the control of midwives and nurses should remain in the hands of the medical and nursing professions, and that this question of registration should be dealt with solely by professional authorities themselves. The Association, therefore, submits this scheme to the General Medical Council, and respectfully seeks the advice, support, and assistance of that influential body in the matter." I shall have, later on, to call attention to the governing body of this Association, in view of the desire that the control of the profession of nurses should remain in the hands of the medical and nursing professions, and to point out presently in what respects the charter fails, speaking of its provisions as being likely to be beneficial to the profession and public at large. At page 65 is the answer of the General Medical Council. A resolution was agreed to: "That if any department of Her Majesty's Government were constituted controlling authority in relation to local managements made under statute for the licensing and registration of midwives, the Medical Council would, if the Government department so wished, be willing to advise as to the general rules of education, examination, and discipline which ought to be established in the matter; but the Council would not be able to discharge, and would, therefore, not be prepared to undertake, any duties of detail as to the registration of midwives, or as to the local arrangements for licensing and controlling them." Then, "On the motion of Sir John Simon, seconded by Dr. MacAlister, it was agreed, 'That in the opinion of the Council it would be much to the advantage of the public, and particularly would be of much convenience to the practitioners of medicine and surgery, that facilities usable under proper guarantees in all parts of the United Kingdom should be given by Act of Parliament or otherwise for the authoritative certification of competent trained nurses, who, when certified, should be subject to common rules of discipline, but that it does not appear to be within the province of the Council to propose legislation or other action for the object referred to, nor has the Council had any occasion to consider in detail the means by which the object might be attained, and that under these circumstances the Council cannot give any opinion on the particular question asked of it in paragraph 5 of the memorandum of the British Nurses' Association.'" Then, my Lords, in April, 1892—your Lordships have got the document before you—the Parliamentary Committee of the British Medical Association passed this resolution. It has been furnished to your Lordships, and it is only given to me in the same way that Sir Michael Hicks Beach's letter was given to my learned friend. This, I understand, is the resolution of the Parliamentary Bills Committee of the British Medical Association.

Sir Horace Davey: That is not the Medical Council. That is a private body.

Sir Richard Webster: It is the Parliamentary Bills Committee of the British Medical Association.

Sir Horace Davey: But that is not an association of any authority—it is not the Medical Council.

Sir Richard Webster: It is not the Medical Council, and I made a mistake if I so stated it; but when my learned friend, Sir Horace Davey, says it is not of any authority, it only shows, perhaps, the spirit with which my friend's instructions are given.

Sir Horace Davey: All I mean is it is a private association of a very restricted character.

Sir Richard Webster: Whether it is a private association of a very restricted character or not, it is certainly not more restricted in character than the present petitioners.

Sir Horace Davey: I dare say not; but you said it was the Medical Council, which is a known statutory body of quite a different character.

Sir Richard Webster: I corrected that, and I quoted it by inaccuracy.

Lord Hobhouse: You are bringing it in as showing the opinion of a number of medical men?

Sir Richard Webster: Yes, I am.

Lord Hobhouse; It does not go beyond that?

Sir Richard Webster: I should be prepared, if necessary, as I have said already, to support this also by evidence. The resolution is as follows: "That, in the opinion of this committee, any register made under the authority and with the sanction of the Privy Council, should be made subject to due regulation and control and with adequate safeguards against the inefficient education or arbitrary exclusion of the persons registered, and with means of protecting the public against the false assumption of registered or registrable titles; and, further, that such register should be made by a representative council of the educating bodies." I am prepared to argue to your Lordships that on principle it is very desirable that those safeguards should be inserted particularly to protect the public against the false assumption of a registered or registrable title, and against inefficient education or arbitrary exclusion from the register. Then following that up, I call your Lordships' attention to page 73 of our case, where we have the objects of the company, which was proposed to be formed without the use of the word "limited"; and I call your Lordships' attention to object 8 on page 73. "To form, control, and carry on—a register of trained nurses: a register of certificated midwives, and to determine from time to time what tests shall be specified by candidates for registration, as evidence that they possess necessary skill and knowledge in their profession." So that when they went to the Board of Trade they proposed to the Board of Trade, perfectly properly, to say we are going to make our register an authoritative document, upon which the public can rely; and I say, at the risk of repeating myself, that if they do not want that, if they only desire to make a directory, it ought not to be a directory by Royal Charter, and they certainly do not require the protection, or the sanction, or the certificate of a Royal Charter for any such object. Then, my Lords, there are two more extracts to show that they have carried the same view down to date. There is the preface to the Register of 1891. "One of the objects for which the Royal British Nurses' Association was founded was to remedy these undoubted abuses by the institution of a system of registration analogous to that enforced by law for many years past for medical men. The following pages are the first-fruits of thre years' incessant work and organisation in this direction. Mistakes may, possibly, have been made, but no care has been spared to reduce the chances of error to a minimum. It must be explained that, in accordance with all precedents, women who had been for three years engaged in nursing the sick, whether trained in hospitals or not, were for the first six months held to be eligible for registration. At the end of that 'period of grace,' three years' hospital service was made an essential condition, and will henceforth be the rule." I am only carrying it on to show your Lordships that down to the actual presentation of the petition, the object I am indicating has been the scheme and object of the Association.

Lord Hannen: I suppose that explains how all these nurses whom you say do not appear to have had three years' qualification got on—namely, that they had been engaged in nursing for three years.

Sir Richard Webster: Oh, no, not for three years, but allowed to get on whether they had worked for three years or not.

Sir Horace Davey: Oh, no.

Lord Hannen: At present that has not been shown. I thought I had got at the explanation of it.

Sir Richard Webster: I am afraid your Lordship has not. The resolution was to require for fresh names three years, but they allowed persons to be registered—Mrs. Bedford Fenwick said so in evidence—who applied before June 13th, 1890, whether they had worked for the three years or not.

Sir Horace Davey: Really there ought to be no contest about this. It is so stated in our books, that during the first six months which is in accordance with precedents—they can be given—registration was open to women, whether trained in hospital or not, who had been engaged for three or more years in nursing the sick, provided their credentials and testimonials were satisfactory to the Registration Board.

Sir Richard Webster: If it is merely a question of being engaged for three years in nursing the sick that may possibly account for it, but I did not understand your Lordship was calling attention to nursing the sick.

Lord Hannen: Yes, I was. I was calling special attention to those words I have marked, "For three years engaged in nursing the sick."

Sir Richard Webster: I have no means of answering your Lordship's question. I will take it from my learned friend, or it can be dealt with in evidence before your Lordships. I have no means of knowing what steps were taken by the Council to see that the persons who applied had been engaged for three years in nursing the sick. That is not material, but that does not deal with many of the other cases I will refer to.

Lord Hannen: I had been waiting for an explanation how all those persons came on the list, and I found it in those words as I thought.

Sir Richard Webster: Not keeping my mind on one particular point I was not following that for the moment. I thought they were let in because, as in many other cases, where membership has been made to depend on qualification—for instance the accountants, the surveyors, and the engineers, and I think I can give your Lordships half a dozen other instances where the charter was going to establish an examining standard—they did not compel existing practitioners to adopt that standard. I will take it at present that there was the qualification of three years engaged in nursing the sick and that may have been the reason, but that of course would not remove the objections that have been raised as to their appearance as trained nurses on this register. A similar statement occurs in the preface to the Register for 1892: "In order to remedy these abuses, the Royal British Nurses' Association began, in 1890, a system of registration analogous to that which has been for many years compulsory upon members of the medical profession. During the first six months, in accordance with precedent, registration was open to women (whether trained in hospitals or not) who had been engaged for three or more years in nursing the sick"—those are the same words, and probably that is the explanation—'provided that their credentials and testimonials were satisfactory to the registration Board appointed by the Association. At the end of that 'period of grace,' three years of hospital training was made an essential condition of registration, and is now the rule. The Board has carefully investigated the credentials of every applicant for registration, and has the power of removing from the register the name of any nurse who, after inquiry, may prove herself hereafter to be unworthy of trust." I am not going back on that point, because it was sufficiently referred to on the previous occasion, but it is practically admitted by Mrs. Bedford Fenwick, in her evidence, that they could not get a person off unless in rare cases people were prepared to come forward and give legal testimony.

Lord Hannen: Would not that be within the scope of bye-laws to determine who should have the power of striking their names off the list?

Sir Richard Webster: As a term of membership, but in no other sense.

Lord Hannen: From their point of view that is all that is necessary, because they say: "We only keep a register of those who choose voluntarily to join us." They would be subject to a certain body who would investigate matters, like the committee of a club.

Sir Richard Webster: I should not be justified in going back on what I have said, but I ask your Lordships respectfully to remember that the object put forward by Mrs. Bedford Fenwick, which I have read to your Lordships this morning, is different.

Lord Hannen: Some are of a different opinion to Mrs. Bedford Fenwick.

Sir Horace Davey: I do not appear for Mrs. Bedford Fenwick.

Sir Richard Webster: My learned friend says he does not appear for Mrs. Bedford Fenwick, but I think it scarcely graceful to disclaim the part that Mrs. Bedford Fenwick has had in promoting this application.

The Marquis of Ripon: All the nurses upon this register are not necessarily members of the Association?

Sir Richard Webster: No, they are not.

The Marquis of Ripon: All the nurses that are members of the Association are on the register.

Sir Richard Webster: I will show that to your Lordship at once. There are 2,813 members of the Association. Every member of the Association is entitled to be put on the register as a trained nurse, but persons are allowed to go on this register without being members of the Association. That is why I ventured to say to Lord Hannen a moment ago, because I have had little experience of these matters, that if it is made a condition or term of membership there is no reason why a committee should not have the power to expel; and what your Lordships have required for the last last six or seven years is that a committee expelling should give an opportunity to the member of being heard before them before his name is removed, but that only applies to members. At present, of course, we have no knowledge at all of what bye-laws might be proposed, but I will assume that the bye-laws would be sanctioned by your Lordships in accordance with precedent for more than twenty years. But this register does not purport to be a register of members; and that is why I did think it important to point out to Lord Hannen that they do not publish a mere register of membership, and will not effect their object if they do, unless they compel every single nurse who desires to appear as a trained nurse to join the Association.

Lord Oxenbridge: Is not it a fact—I have always understood so—that it takes much more time frequently for the training of a nurse in a hospital to what it takes in a training establishment?

Sir Richard Webster: I think so.

Lord Oxenbridge: I know a very celebrated training establishment where they only have them for twelve months, and they frequently go out—that is, at a cost per head of £50 in that training establishment. How are they to be got on the register if the qualificatian is three years?

Sir Richard Webster: They cannot possibly be got on it.

Lord Hannen: Then it becomes a register of three-years trained nurses.

Sir Richard Webster: Instead of being a register of trained nurses.

Lord Hannen: It can be altered by a word.

Sir Richard Webster: Yes, but there has been no offer to alter it by a word before your Lordships. Your Lordships can unquestionably make a register which will possibly prevent the chartered body from gaining any one of the objects which was desired. The question is whether it should be by charter. Your Lordships will not lose sight of the point I have made, that I know of no case where the object of a chartered corporation has been to keep a register, and that is a great deal more than making a register by bye-laws of members. The object of the corporation is to keep something. That would mean that her Majesty thinks, on the advice of your Lordships, that the chartered register is to be not optional, but what the Association are to keep. My Lords, with regard to the observations made by Lord Oxenbridge, I will call your Lordships' attention to paragraph 451 of the third report of the House of Lords' Committee: "Different opinions are held as to the length of training requisite before a woman should be sent out with a certificate as a trained nurse. A witness who had had experience as Matron of St. Bartholomew's Hospital, was of opinion that nothing less than three years should be taken as the qualifying period, and that no woman ought to be made sister of a ward or staff nurse, or be sent out to nurse the sick, until she has passed through the whole curriculum."—Your Lordships will remember you were told by my learned friend, Sir Horace Davey, that the greater part of St. Bartholomew's Hospital was supporting the petition.—"Miss Nightingale, on the other hand, has laid down one year as the ordinary period of training, with the proviso that it would be preferable to give two years' training to those who will have to train others in their turn. At St. Thomas's, where the nursing is organised according to Miss Nightingale's system, the probationer, after her month's trial, binds herself to hospital service for four years; after one year, if she passes her examination, she is registered as a certificated nurse, and thereupon, for another three years, she holds herself at the disposition of the Committee of the Nightingale Fund for Hospital Nursing. At other hospitals the engagement does not extend beyond the period of training, but that period is prolonged to two or three years, so that the hospital, after it has trained the nurse, may still have the benefit, for a time, of her trained services, the longer period being fixed rather for the sake of increasing the nurse's experience"—this bears directly on what his Lordship (Lord Oxenbridge) said, and is confirmed by the information given me by those who would give evidence before your Lordships—"and for the convenience of the hospital than from the belief that she would not be fit to receive a certificate sooner. At the London Hospital, for example, a nurse is certificated after two years' service, but is, in some cases, given the duty of a fully-qualified nurse in the hospital, or sent out to nurse a private case, occasionally is even appointed to be a sister of a ward, while still called a probationer. Length of service is only one of several elements which go to make a good nurse; and the opinion was strongly expressed that more reliance was to be placed on a system of careful individual supervision and selection than on any extension of the probationary period. At the London Hospital, out of about 210 sisters, nurses, and probationers, fully one-half (including about 50 probationers in the second year) were regarded as qualified nurses." Then, my Lords, at page 392 of the first report occurs the passage in the evidence of Miss Lückes, which I had in my mind when I told your Lordships some time ago that the period required was by no means as long in all cases. This is the evidence of Miss Lückes, as to whom Sir Horace Davey very fairly gave testimony the other day, upon his instructions, as to the work she had done, and her great experience in this matter. "(6,633). As to certificated nurses, you say that uncertificated probationers are sometimes put as acting sisters in command of certificated nurses? *A*. That is so sometimes. I think some confusion arises from the fact that Miss Yatman ascribes an imaginary value to the certificate. (6,634). You consider, then, that uncertificated probationers are sometimes more versed in nursing than certificated nurses? *A*. Very often. (6,635) You do not think that the system of putting certificated nurses under the charge of uncertificated ones causes friction? *A*. I never had a case of it. Of course the one who is acting as sister must be superior."

The Marquis of Ripon: You need not go into the particular case.

Sir Richard Webster: No, my Lords; with regard to this question of legal evidence, Lord Hannen put to me that which I have never concealed from myself, that as far as the members were concerned, they could, of course, be got off the register, but we have got to deal with the persons who are not members, and I will call your Lordships' attention to Dr. Bedford Fenwick's evidence with reference to this matter, at page 757 of the second report: "Suppose I wrote to you to tell you that Nurse So-and-So had been drunk on a certain occasion on duty, would a communication of that sort be actionable? *A*. We should ask you to be good enough to verify your words. (26,056) What verification would you ask for? *A*. We should ask you to be good enough to give us the opportunity of acting legally upon it. (26,057) Then it would render me liable to an action if the thing were not exactly proved? *A*. We should take it for granted that you would be able to prove your statement." Then there is a question abous the degree of drunkenness, and then Dr. Bedford Fenwick says this, "That is a difficulty, but we should hope the public would, for its own sake, tell us the facts, and be willing to support their statements." The other evidence with reference to its being impossible to revise this list in the sense of finding out all the persons no longer fit to be on the register is referred to in the marginal notes at page 90 of the third Report, paragraph 512: "A point very strongly urged is that the character of the woman herself is a most essential matter in regard to a nurse; much more so in the case of a nurse than a doctor. The Association professes to require evidence of character (by the production of recent testimonials) before it will put a nurse on its register, and to register only women who have had three years' hospital training; but it appears women are registered who have not completed their full period of training at any one hospital, and of whom it is not known whether they have proved themselves competent or otherwise. The Association complains that a hospital certificate once given cannot be withdrawn, whereas a name will be removed from the register whenever a nurse is proved to have forfeited her good character, legal proof being admittedly exceedingly difficult. But it is evident that this course cannot be taken except on clear proof of actual crime or misconduct, and therefore it is no protection to the public from mere incompetency. It was admitted that a woman might go through three years' training at a hospital and get her certificate, and yet be a very indifferent nurse, and be known at the hospital to be so; but the public who read her name in the register would suppose her to be competent, unless the register clearly stated that it did not guarantee the efficiency of its nurses. On the other hand, if the Association disclaims responsibility for the efficiency of the nurses whom it registers, it seems difficult to understand wherein lies the security which it offers to the public." My Lords, one might test that by asking whether the Association would like it to be at the head of every page of their register: "The Association is not responsible for the efficiency of the nurses whom they register." It is obvious if that was so the chartered register would not have any of the effects which those who instruct me naturally feel the register would have, used as it must be used. The Association actually say they are not responsible for the hospital training, but say that they have carefully examined the credentials of every applicant, and have the power of removing from the register the name of any nurse who, after full inquiry, shall prove herself to be unworthy of trust. Therefore, they put it forward on the face of their advertisement that though not responsible for the hospital credentials, they are responsible for the trustworthiness of the nurse whose name is on the register. My Lords, I have now to call your attention to another great defect, so far as the constitution of this Association is concerned, with regard to the persons who are contemplated by the charter to be its members and to be the governing body. I will ask your Lordships to be good enough to look at page 6 of the charter.

Lord Hobhouse: You are going to the constitution of the body?

Sir Richard Webster: Yes. Now, my Lords, they propose that the only members shall be medical practitioners and nurses. My Lords, that excludes without exception, perhaps, persons who know as much at least, and possibly more, about this question of training than anyone. The training schools of the hospitals have been managed by lay managers and lay members of the training committees who have given up an immense amount of time in many instances, and are of great experience. In the opinion of those who instruct me, and I certainly have every right to ask your Lordships that weight should be given to their views, in the matter of the training of nurses the experience gained by the lay managers, apart from the mere question of medical knowledge such as doctors would have, is of the greatest service in judging of what nurses have effectively, and properly, and successfully carried on the practice of their profession. My Lords, in London there are twelve hospitals which have training schools attached to them. Giving those for whom my learned friends appear credit in any case in which they have partial support, and not attempting to divide the support where it is not unanimous, but assuming partial support means unanimous support—though that is not the case—there are four hospitals in London supporting my learned friend, and eight opposing the present application with reference to the register, and what is most significant is that if you take it by number of beds there are 1,540 beds in the four hospitals in favour as contrasted with 2,751 in those against. So that it cannot by any means be suggested that the institutions who have the most experience in training are supporting my learned friend. Then, if you go to the provincial hospitals, again there are seven provincial hospitals supporting the petition against eleven opposing it, and in every case there has been included amongst the seven those who only give a partial support, though in many instances distinguished members of the body are against the petition. It amounts to this, that speaking of it as a profession, there are altogether thirty large nurse training institutions of which nineteen are opposed to this register, as against eleven who are wholly or in part in favour of it, and there are some 6,000 beds included in the nineteen institutions showing the size of the schools, as against 2,622 beds in the eleven hospitals in favour. Therefore, I point out that not only is the support not that of the majority of those skilled in training nurses, but there is excluded from the governing body who ask by this charter to be constituted into the authority for nurses those gentlemen and ladies who, by experience and knowledge in connection with the training schools would be best competent, or amongst those best competent to consider what is a proper qualification. Now, my Lords, this is not a mere accidental omission. I read to your Lordships in the few moments when Lord Ripon was out of the room that the object of the Association was that the control of the nursing profession should be in the hands of medical men and nurses. Therefore, this is a point which we are really entitled to press before your Lordships, because this is a point which cannot be altered. No bye-law can alter this. This will be in the charter, and an essential condition of the charter if it stands substantially in the words in which it has been presented. Under the circumstances the members from whom the majority of the governing body must be chosen will be entirely medical practitioners and nurses, and it is significant to know that the far larger majority will be nurses, so that if the ordinary principles of voting prevail the nurses will be governing themselves without any outside influence or authority. My Lords, there is one main point with regard to this qualification that is not immaterial as showing your Lordships the extreme importance of preventing any institution from directly or indirectly suggesting that their nurses are the only trained nurses. My Lords, I believe a Royal charter has been already granted to the Queen Victoria Jubilee Institute, and that does not provide for a register of nurses as having authority of any kind, and as regards that Institution nurses are entitled to become members after one year's training. They are allowed to be called "Queen's Nurses," and they have a badge, which they are entitled to wear as long as they fulfil certain conditions. I do not know myself the exact particulars, but they can be furnished to your Lordships by the officials if necessary. It certainly is a most remarkable thing that these Queen's nurses, sanctioned by Royal charter, and having privileges given them, would not be entitled to get upon the register now proposed, so that they must be excluded from the register of trained nurses. It is not a question merely of the Association of Trained Nurses, but the sting of the thing is that it is a register of trained nurses for 1892, meaning that details shall not be required as regards trained nurses, and that those who have not the advantages of being registered as trained nurses will not gain the custom or get the highest scale of remuneration.

Sir Horace Davey: The nurses are not members.

Sir Richard Webster: Then nothing could be a stronger illustration of the force of my point, that they, by their notices, are putting out to the public that they are members whom they choose to include among them on the register of trained nurses for the year 1892. Here are chartered nurses, if that is the proper expression, who are authorised to act in that capacity, and to have certain privileges under and by virtue of a Royal charter, the object of which is the maintenance of a fund to be applied for the support and training of women for the nursing of the sick poor. My Lords, I must protest against the attitude of the present opponents to this application being misrepresented or distorted. We are not here to object to a charter which will confine itself to proper objects, as, for instance, the collection of funds, or the improvement of nurses, or the establishment of a college; but we say that, under colour of that application, they are desirous of obtaining a chartered register, which will be the means of their holding out directly to the public that nurses not on the chartered register are not trained nurses, and are not trained nurses on whom the public ought to place reliance. Then, my Lords, it was said that the object of the Association was to guard the public against unqualified nurses being put upon them, because their names would not appear on the register. Therefore, the object is, whatever you may call it, although it may be only called a directory, to obtain for the particular members and nurses of this Association special privileges and special rights. Looking at the "Nurses' Journal," it is a little significant that in 1891 there were 2,829 members, and at the present day, according to the list, there are only 2,532, the number having diminished between 1891 and the present month of November by 297. I take that from the "Nurses' Journal," the organ of this Association. That, my Lords, brings me to another point. Sir Horace Davey quoted the number of nurses who were supporting him, and he assumed that all the members of the Associatioh supported him. That is really not so. The nurses never signed this petition. The gracious lady who s the president signed on behalf of all the nurses, but the nurses themselves have never been asked, as individual nurses, to express their opinion upon this question of a register, and, as a matter of fact, although I do not want to mention names, but I can do so if your Lordships desire it, there are several nurses who are members of the Association who have signed the petition against the proposed charter. There is one other fact I may mention. The signatures of those who support the petition have been obtained by canvassing, and, no doubt, there is great credit due to the ladies who have taken so active a part in the matter. I think it is stated that a few hard-worked nurses obtained these signatures in a comparatively short time.

Lord Hobhouse: I do not think you can get the signatures of a large body of people for any object without canvassing, Sir Richard.

Sir Richard Webster; Probably not, my Lord; but I wish to state that there has been no canvassing of any sort or kind on the part of those whom I have the honour to represent. So that I submit your Lordships, therefore, must not treat the mere enumeration with that amount of authority which Sir Horace Davey desired your Lordships should. More than once on the last occasion Lord Hobhouse said to me, "Have you not got the analogy of the medical register?" I will ask your Lordships' kind consideration of the marked distinction there is as regards that, and of the safeguards which the Legislature thought fit to impose in connection with that register, and I say, subject to correction, that not one of those can be granted by a Royal charter. In the first place, no private individual can be compelled to give information; that is to say, they have no means of obtaining information, nor is there any duty to send information to the governing body either of incompetence or of untrustworthiness, or breach of duty on the part of the nurse. No person sending information will be protected from libel, nor could any charter give that protection. That would be contrary to the law of the realm.

Lord Hobhouse: The medical council is empowered to demand information.

Sir Richard Webster: Yes, my Lord, and notice has to be given. All this was made the subject of statutory enactment in the Medical Act, 1858, and by the 28th section of that Act notice is obliged to be given to the central body if the names of any constituents are struck off. That is 21 and 22 Vict., chapter 90.

Lord Hobhouse: I rather think that is the Act which constituted the Council.

Sir Richard Webster: Yes, my Lord, it is the ruling Act. In this case, in order to make this register effective, it must be the duty not only of every hospital, but of every nursing institution which sends out a nurse, to communicate information to the various bodies if it is to be of any avail. I may at once call your Lordships' attention to the enormous difference there is when you are dealing with the nursing profession as compared with the medical profession. What has been described in the argument before your Lordships as tact, judgment, prudence, and steadiness are admitted to be essential as a medical qualification. Of course, there are degrees of that, and your Lordships sitting here, great as your Lordships' wisdom is, are not in a position to say what a Parliamentary Committee could say after an examination of witnesses either by a hybrid Committee, or otherwise, as to the merits of the case, and such an enquiry the Board of Trade thought essential before such a privilege could be granted. Section 45 of the Act says that notice is to be given by every registrar of the death of every medical practitioner, so that the register may be corrected from time to time in order to establish an effective register. Then, by sections 18 to 23, control is given over the teaching bodies. I need only read to your Lordships the marginal notes of the sections. Your Lordships will observe that "the Council may require information as to the course of study, &c. required for obtaining qualifications." That is to say, the British Association of Nurses ought to have power to send to the hospital and ask what is the course of study they require before they give a certificate. That is most important. It is not denied by my learned friend that the standard of training is very different in many of these institutions. In many of them a year's work will be worth as much as three years in others; and in some institutions it will be admitted that the training is, comparatively speaking, of an insufficient character; so much so, that they would not take it as evidence of a first-class certificate. Then there is a section which enables the examining body to obtain any information as to the curriculum, and the colleges may also unite in conducting the examinations, which, of course, may be a very useful thing, as tending to a standard of uniform excellence. Then it is provided that defects in the course of study may be represented by the General Council to the Privy Council; "and the Privy Council may suspend the right of registration in respect of qualifications granted by the colleges in default," &c. Then the marginal note of the next section is this: "Persons not to be registered in respect of qualifications granted before revocation." No charter that your Lordships could advise Her Gracious Majesty to sanction could impose any such restrictions as that. The only restrictions your Lordships could impose would be that the bye-laws of the charter should be subject to the approval of your Lordships. But your Lordships cannot direct that there should be any particular curriculum, or any control over the training of nurses which the Association are desirous of reaping the benefit of. I call attention to the charter contemplating penalties for breach of the register, if I may so call it. Your Lordships may be aware that by section 38 of the Medical Act the falsification of the register is a misdemeanour, and registration obtained by fraud is made by section 39 an offence. Now just conceive the importance of that. This document is to go forth to the public as the chartered register of trained nurses. The entry on that register may have been obtained by false representation, or by giving a false character. It is stated that the governing body (to quote the actual language) investigated the credentials of every applicant for registration. Supposing false credentials are sent that is no offence, and yet the analogy is put of the medical register, and it is asked, Is it not as important for medical men as for nurses? I say certainly it is if you have a proper and satisfactory method of dealing with the difficulties that have to be grappled with. Then, my Lords, what is to prevent a person passing herself off as being a registered nurse? Everybody will not have this register at their disposal, and the Legislature by section 40 made it an offence for a man to call himself a physician or doctor, and so on, without being duly qualified. I do not want to repeat myself, but your Lordships will find that there are the same provisions practically in the Pharmacy Act of 1868 and the Veterinary Act of 1881.

Lord Hobhouse: Do you mean the same provisions as are inserted in the proposed charter here?

Sir Richard Webster: No, my Lord, I mean the same provisions as are to be found in the Medical Act.

Lord Hobhouse: I understand you.

Lord Hannen: They are all general registers, and a man must be upon the register in order to practise.

Lord Hobhouse: They profess to provide for reasonable penalties.

Sir Richard Webster: But your Lordship cannot do that. Therefore, I say there is no protection to the public at all. It may be an immoral thing to do, but there is no protection to the public against breaches of the regulations. I am not saying that all this is necessary in the case of nurses, but it just the thing that requires to be inquired into, and in the resolution which I read, which my learned friend for the moment was instructed to say was passed by an association not of much weight, it was pointed out that these are the kind of safeguards that ought to be affixed or enforced in the case of any register which is to have anything like authority that a register must have if it is one of the objects of the corporation to maintain a chartered register.

Lord Hannen: You used the words "applicant for registration." Where do you find that?

Sir Richard Webster: I took that from the Register for 1892, my Lord, at page 6, where it says, "The Board has carefully investigated the credentials of every applicant for registration." That is not a member.

Lord Hannen: I infer from that that a nurse's name would not appear upon this list unless she was an applicant herself?

Sir Richard Webster: No, my Lord.

Lord Hannen: Conditions may be imposed on persons applying?

Sir Richard Webster: Something with regard to the question of membership, your Lordship means?

Lord Hannen: Yes.

Sir Richard Webster: It is very doubtful how far such conditions could be enforced.

Lord Hannen: It might be by taking the name off.

Sir Richard Webster: That, of course, would be assuming circumstances to come to their knowledge.

Lord Hannen: Yes.

Sir Richard Webster: But even then, my Lord, there would be no protection.

Lord Hannen: This would need the protection that has been given in these cases by Act of Parliament.

Lord Hobhouse: A new applicant the Association may refuse to register unless certain conditions are complied with.

Lord Hannen: And they might impose the condition that their name would be taken off under certain circumstances.

Lord Hobhouse: Your objection, as I understand it, is that that gives the Association power to fix a test for the competency of nurses.

Sir Richard Webster: I am inclined to think that some such condition could be imposed; but if your Lordship looks at the previous bye laws you will see the jurisdiction over a person who is not a member of the corporation would be different from that over a person applying to become a member.

Lord Hannen: Yes. They might say, "We will not admit you at all unless you comply with certain conditions."

Sir Richard Webster: That, of course, my Lord, is possible. Then, further than that, the lecturers to the nurses are a body whose opinion is entitled to great weight. The instructions to those nurses are given by medical men. I do not want to count heads, but if your Lordships look at the evidence before you, you will find that the vast majority of those who have been instructing the nurses for 18 or 20 years past are against the system of allowing an independent body to give a nurse a certificate for competency. They say (and their view is entitled to consideration) that persons ought to be led to look to the place where the best information can be given. Sir Horace Davey indicated that half a register is better than none. But, my Lords, we submit that half a register is very much worse than none, because half a register will mislead, whereas none will not. The way in which it is worked with regard to hospitals is this—that if persons send to the hospitals for a nurse sometimes the hospital may know the kind of case. But a training school can probably send out a nurse who, up to date, as far as the institutions know, is qualified for that particular case. It is impossible that any such information can be given by a register, or by application to the secretary of such an Association. Therefore, I submit to your Lordships that half a register is worse than none, because if the register contained only half what they ought to know that would prevent people going to the places where they could obtain the information. Lord Hobhouse put to me the other day a question as to what course I should take. I think I should go off to the hospital to inquire; but that does not apply unless the register includes all the nurses, because, *ex hypothesi*, a large body of these people who are skilled in nursing say that there are as good nurses as those who are on the register who cannot be put on the register because of the particular rules. It is important that the public should be led to go to the institution that does know most about the nurses, and not to a register kept by people who do not know as much. On that question I repeat (and I will prove it, if necessary, by evidence before your Lordships) that no application of any kind has been made to any one of the hospitals I represent prior to the placing of their names upon the register, or with a view to the annual revision of the list since the time the register has been published. My Lords, I am afraid I should be unduly trespassing upon your time if I reminded your Lordships in detail of the overwhelming testimony which there was before the Lords' Committee as to the importance with regard to the practical work of knowing how a nurse had carried on her duties in the hospital. I think your Lordships will agree generally in the force of that argument. It has been suggested that if you regard this simply as a list where the public can obtain information that will be the means of enabling you to get that information. I have sufficiently dealt with that, and should not be justified in occupying your Lordships' time by reading the evidence of Mr. Bonham Carter, or of Miss Lückes, and other persons, including Dr. and Mrs. Bedford Fenwick, with reference to the extreme importance of these characteristics, and the admissions made by them that those are matters that cannot appear, directly or indirectly, upon the register. My Lords, I do not think that there is any other separate head which I desire to press upon your Lordships. I have in this case the advantage of the assistance of my learned friend, Mr. Bristowe, and I trust your Lordships will permit him to address you on this matter.

Lord Hobhouse: We do not hear more than one counsel on each side in these cases.

The Marquis of Ripon: After your address to us, Sir Richard Webster, I am sure we shall not need any further assistance.

Sir Richard Webster: I am much obliged to your Lordship. Then your Lordships will allow me to say this. You will have gathered that hospital experience, and not training, is that which the Association make the condition. That bears upon what I said just now, that the certificate is not a certificate based on three years' training or anything of that kind. The first regulation on page 8 is this, "Applicants for registration must produce the proof that they have been engaged for three years in work in hospitals or infirmaries." That may mean anything. That is most elastic phraseology, "Engaged for three years in work in hospitals or infirmaries."

Lord Hannen: It must mean hospital work. You would not suggest it meant sweeping the floor.

Sir Richard Webster: No, my Lord, I do not suggest that. But, my Lords, it was suggested it meant certificates of high training, and that it was opposed by us because some of those whom I represent desired a less period. As a matter of fact there is on the register an instance of a person who was only a nursemaid in the children's ward. I did not put it quite so low as anyone sweeping the floors, but your Lordships will find that stated. I do not know that I should have referred to that but for your Lordships' very natural observation with reference to my statement. My Lords, I will not trouble your Lordships further, I desire to submit, on behalf of the vast majority of those who have been engaged in the active training of nurses, both laymen, matrons, lecturers, and a very considerable number of medical men, that, without full inquiry as to what is the natural effect of a such a register, and as to the way in which such a register can be safeguarded, no corporation ought to be created by charter to have as its object the keeping of a register of trained nurses. If the charter, at page 4, had omitted to state that the purpose of the corporation was the maintenance of a list or register of nurses, which is wholly unnecessary if it is merely a list of members, because that could be provided for by bye-laws, then very different considerations might have arisen. There are those who have been labouring for years in this matter who are not averse to a proper register being kept by a responsible body, but they desire me to state that if it be safeguarded by such provisions as the Legislature in its wisdom think should surround such a document in order to give it authority and to give it sanction, and to give it that *imprimatur* which the public are entitled to expect of it, then they would welcome it. But in order that it may have that effect it is essential that persons who have the training of nurses should have a large share in considering and determining the way in which a register should be prepared, and the way in which it should be kept authentic. It is necessary that there should be statutory provisions to enable the managers of this register to do their work in the interests of the public. We humbly submit that if what they now desire falls short of that, then they do not want it as one of the purposes of their charter, and what ever your Lordships' opinion may be as to the granting of a charter, at least it should not be in the power of the managers of this institution in future, apart from your Lordships' control, to hold out to the public that that register will be a register of trained nurses so as to give the nurses registered by them an advantage over their sisters which they have no claim to, and to prevent persons equally talented and equally qualified, but who have not happened to fulfil the conditions of the Association which may vary from time to time from carrying on their business with the same effect as they might have done if they had become members of the Association. I am sorry to have had to address your Lordships at so great a length, and I should not have done so had it not been regarded by Miss Nightingale and those who have laboured for years in improving the status of nurses, and who are continuously engaged in the work as a most important matter. They think the publication of a Chartered Register by the British Association of Nurses to be in the hands of doctors and nurses alone would lower the status of a nurse, and make it less likely that she would be fully qualified, and that, further, it would impose impediments in the way of talented and experienced nurses, which, if they are at all justified in their apprehension, would not, I am sure, meet with your Lordships' approval. Then, my Lords, I ought to mention this. If your Lordships think it right I desire to call evidence on one or two points. I have the witnesses here, and tender them. Perhaps your Lordships will kindly consider that. I ask to be allowed to call evidence if your Lordships think that the procedure here will admit of it.

Lord Hobhouse: On what point do you think it desirable to call evidence?

Sir Richard Webster: I should propose to call evidence to show that the mere standard of three years' work in a hospital is not by any means, under any circumstances, a satisfactory condition of the person being advertised as a trained nurse; secondly, to show that many persons have become quite skilled and qualified with less training; and, thirdly, the fact that the hospitals have remained in communication with their nurses for years afterwards, and obtain information as regards their personal capacity for work. Then I should desire to prove that no application has been made to the hospitals by the Association with regard to any information as to putting the nurses' names on the register, excepting in one or two special instances with regard to their removals. I am not, of course, suggesting that it has been your Lordships' practice to take evidence in such matters.

The Marquis of Ripon: In my experience I do not recollect such a thing.

Sir Richard Webster: Nor do I, my Lord. I only desire to enforce what I have said, by stating that if the merits require to be investigated before such privileges should be given, some tribunal should investigate them, and if your Lordships are prepared to go into that inquiry, we have the witnesses here whom we can call.

Lord Hobhouse: My impression is that the controversy as to the points which you have mentioned will not very much affect the matter. There is a great deal of evidence before us upon that point already. You have called our attention to the statements of the witnesses who were called before the committee on every one of those points, I think.

Sir Richard Webster: Yes, my Lord. I only intended to say that my clients desire, if your Lordships feel you can hear the evidence, to place it before you. After all, the statements of counsel, of course, are only made upon instructions.

The Marquis of Ripon: We will consider the matter during the adjournment. I must say that I do not myself see any reason for departing in this case from the ordinary course.

Their Lordships adjourned for a short time.

The Marquis of Ripon: I may say before you proceed, Sir Horace Davey, to reply, that their Lordships have decided not to hear evidence in this case.

Sir Horace Davey: I am much obliged to your Lordships for that intimation. Now, my Lords, I desire in the first place to point out exactly the way in which the argument stands at the present time. If I understand my learned friend's argument last week, and also to-day, correctly, my learned friend is not instructed to say that the other objects and purposes of this Association are not such as deserve approbation and commendation on the part of the Crown, and are such as will reasonably entitle the Association to crave the status of a corporate body.

Sir Richard Webster: That is perfectly right. It is for your Lordships, of course, to consider that. We did not address any argument upon that point.

Sir Horace Davey: I am glad that my friend has interrupted me, because it shows that I understand his argument correctly. Nor did I understand my friend to say, having regard to various precedents, some of which were mentioned incidentally to your Lordships, that of the Surveyors' Institution and others, that this Association was not a proper association to receive the honour and favour of incorporation at the hands of the Crown, subject, of course, as I agree, to the discretion vested in your Lordships advising Her Majesty. But then my learned friend says that "in your draft charter you seek to have it declared that it is a purpose of your Association to keep a register of trained nurses." It is to that point my learned friend Sir Richard Webster has directed the full force of his artillery. Now, my Lords, let me clear away one or two matters of fact in the first instance before I devote myself to what I conceive to be the solid ground of argument that has been used by my learned friend. Sir Richard Webster, speaking, of course, from instructions, has said, for instance, that the number of members of this Association has declined during the last few months. I have enquired of the gentleman who represents the Association, and I find that that is the fact, and, my Lords, I am not surprised that that is the fact when I regard the amount and weight of the opposition which they have encountered, the quarters from which it has proceeded, and the manner in which it has been conducted. The amount of misapprehension—I carefully use that expression—as to their motives, objects, aims, and methods, has been such as to expose them to a continual storm of opposition from the moment of their existence, and, my Lords, I regret to say that that misapprehension has not ceased, even at your Lordship's Bar, because my learned friend was instructed to say (and I am quite sure on a point of that kind he would not go one hair's breadth beyond that which his instructions warranted) that when the Association registered nurses they never applied to the hospitals. I have been furnished with the circular letter which they sent both to the referees, and also to the hospitals where they have been trained, and whose certificates they held. I do not wish to make it a personal matter, and I will read only one of the forms. It is headed "Royal British Nurses' Association," and it says: "Madam (blank) has applied for registration, and has presented a certificate of efficiency issuing from (blank) I am directed by the Registration Board to ask you to do me the favour of giving me what information you can concerning this nurse."

Sir Richard Webster: When was that sent out?

Sir Horace Davey: That has been in use, I understand, during this year. Before that a letter—not a circular—used to be written for the occasion.

Sir Richard Webster: I only ask because I do not think it was even in this year.

Sir Horace Davey: I am told by the gentleman who represents the Association that that has been sent out ever since last March. Now, my Lords, you will be surprised to hear after what my learned friend has said that from three of the great hospitals we never got an answer to our application. It it is quite true that my learned friend may be instructed to say that they never refused us information. Speaking by the card, which I am quite sure my learned friend would not do, that is true—that is to say that no letter can be produced in which they refused it, but they simply took the course of ignoring the application and not answering it at all.

Sir Richard Webster: The register of 1892 was issued in he earlier part of this year before this circular was in use. should like to see any previous letter. I am told that no uch application was made.

Sir Horace Davey: But you would have the letters.

Sir Richard Webster: We could not have the letters if they were not sent.

Lord Hobhouse: Is that very material, really? If there have been any shortcomings of that kind we should not refuse you a charter on that account.

Sir Horace Davey: But, my Lords, I should not like it to be thought that the ladies and gentlemen who instruct me have been so defective and so deficient either in courtesy or in fairness (whatever we may think of our opponents), or in right dealing I should call it, as not at once to communicate with an institution of which a certificate is placed before them as a credential. Of course, if these institutions do not think fit to reply, and to ignore the Association, they are at perfect liberty to do so, but they are not at liberty to take that course and then to complain that they have not been consulted.

Lord Hobhouse: This point struck me really as the only one of those upon which it was suggested evidence might be given, which really is in controversy, but I think nothing could turn upon it. We might have endless squabbles and that would not affect the judgment of their Lordships which must be founded on much more vital points.

Sir Horace Davey: Quite so, my Lord but I am anxious that the ladies and gentlemen whom I represent should not labour under the imputation of taking a course which, in my opinion (and I say it deliberately) would be contrary not only to fairness but to right dealing, and would disentitle them to any consideration at the hands of your Lordships or of anybody else.

That, my Lords, being the position in which the argument stands, I am at a loss to conceive why we should not have our charter. Whether any limitations should be imposed upon us, or any restrictions should be imposed upon the corporation to be created, is entirely another question, but I

repeat that so far as my learned friend's argument goes to the general question, whether or not the Royal British Nurses' Association should be granted a charter, not only we have not been met with opposition on behalf of my learned friend, but if I understand him correctly he says that it is a proper case for a charter. What, therefore, is my learned friend's contention? Does he wish it put into the charter that we are to be prohibited from keeping a list or register in which persons may voluntarily register themselves? If so, that would be one of the most extraordinary provisions I ever saw, because in order to make my friend's argument intelligible, and to carry out the idea that runs through it all—it should contain a provision which would prohibit us from keeping or publishing any such register. This is an answer to a great deal of my learned friend's argument, and the opinions which he read of persons of whom I shall speak with most absolute courtesy and consideration. I will only say that those opinions are founded on a misapprehension. I agree—and I will go further and say I cordially agree—with a great deal which my learned friend has said. I agree with him for instance, as to the mere technical training of nurses not being sufficient to give a guarantee of excellence either of the character or of the competence of a nurse. We must all agree with that, but you may say exactly the same thing of the register of dentists or the register of doctors, and in a less degree of the register of chemists. I am sure that any of us who have ever suffered, or shall I say benefited by the hands of that admirable section of the medical profession, will agree that softness of touch, delicacy of feeling, sympathy, and gentleness, and general manners, are exceedingly essential in the making of a good dentist.

Lord Hannen: Have the dentists a charter?

Sir Horace Davey: No, my Lord, but they have got a register by Act of Parliament. You cannot register all those qualities on a dentists' register. But, my Lords, it may be a condition precedent that there should be a register expressing the minimum limit of qualification, but if you want a good dentist you trust to reputation, and to the recommendation of your friends, or you may trust to recent experience, or if you have, happily for yourself, had no previous experience, then you must trust to the general reputation a practitioner has earned, or to the recommendation you receive from your friends. It comes to this, that you must in the long run, if you are to select a person, trust to his private reputation and to private recommendation, and to information generally of that character. Now, my Lords, is that any objection to there being a register in a case of this kind? I trow not. It is quite true the register will not tell you that, but it will tell you that the person who is on the register has presented credentials of good conduct to the Registration Board who have put a particular nurse on the register, and has presented a certificate of having been at a certain hospital or training school. My learned friend says that those certificates may be forged. Of course they may; but the best means of detecting forgery is to communicate with the hospital to see whether they are right or not.

Sir Richard Webster: Certificates of character may also be forged.

Sir Horace Davey: You can only make inquiries of the person who gives them, and each person has to give three referees. Certificates, of course, may be forged now, and there are no means of detecting them. A nurse may bring to me a certificate purporting to come from St. Thomas's Hospital, and except by communicating with St. Thomas's Hospital, I have no means of knowing whether it is genuine or not. But if I have the register I have a body who has done it for me—a body who has ascertained, as far as it can, whether that certificate is a correct one or not, and who would not put a person on the list until the genuineness of that certificate had been ascertained by inquiry at the hospital. It would be their obvious duty—and a duty which I am quite sure the persons whom it is proposed shall form the Registration Board of this Association, and who do form it at present, would perform—not to neglect to take the best care, and the best means, open to them to ascertain the genuineness and sufficiency of the certificates and credentials, whether of efficiency or of character. I venture to think, my Lords, that that is no objection.

Then, my Lords, my learned friend has expressed some mild surprise, rather perhaps, in a forensic manner, that I had been unable to grasp the reasons why a voluntary registration of this description was injurious to the interests of, it is said, both nurses and medical men, and also the public. For the purpose of establishing that and supplying my unaided intelligence and your Lordships with the reasons for it, my learned friend quoted from the evidence of Mr. Treves, who is a surgeon and instructor in one of the hospitals—I think the London Hospital. I was very much obliged to my learned friend for doing that, and I was much interested in reading the reasons which that gentleman gives. No doubt that gentleman is one of the highest competency, and one who is well instructed in the subject, and who has thought about, and is well acquainted with the details of it. I take him further as the one my learned friend put forward as the exponent whom he chooses of the views he expresses. I am not going to read again his evidence, unless your Lordships wish it. It was read by Sir Richard Webster at some length the other day, and your Lordships will find it at question 7,754, page 457, of the first report of the Lords' Committee, That gentleman purports to give his reasons why it is injurious both to the nurses and medical men, and apparently (although I do not quite understand what he says) his answer on that point, which is to be found at the top of page 458, is that the register would reduce all the nurses to one level, and that in his opinion the training colleges and hospitals ought to have the monopoly (he does not use that expression, but that is my paraphrase of his statement) of supplying nurses. My Lords, that is exactly what we say, that this case is a case in which the nurses and the medical men ask to manage their own affairs. They ask not to be under the complete supervision and complete control which apparently (and I only say apparently), the hospitals and training schools desire to keep them. Your Lordships will observe what the gentleman says as to the nurses having all been under a certain curriculum. It really comes to this—if you want a nurse for fever, or small-pox, or any other particular form of malady to which we are subject, or a difficult surgical case, then, no doubt, you will get the best nurse through inquiry at the hospital. But, my Lords, that is not the question. The question is whether the number of nurses who are not on the books of the hospital, and who have no means of obtaining a recommendation from a hospital or training school, are to be excluded from the exercise of their profession, or, in other words, whether the hospitals are to have the exclusive right of supplying or furnishing the public with nurses. What this gentleman apparently means is, that opening such a voluntary register as we propose to open would be injurious to those favoured nurses who are sent out by the different hospitals. Then, my Lords, what does he say with regard to the public? He says it would be injurious to the public, because all the nurses would be on a dead level, and you would get nurses, if you took them from a register merely, without any knowledge of their qualification. This gentleman may be a most excellent surgeon, and I have not the slightest doubt he is; but he appears to me to have very little knowledge of the world, and still less power of ratiocination. That is founded on a misapprehension. We do not put them all on a level. All we do is to register their names and to say these persons have fulfilled certain qualifications. That is all we can do. As to saying everybody who has fulfilled that qualification is on a level with everybody else that is preposterous. But, my Lords, unfortunately that gentleman gave an illustration, and he said that if this were a register of governesses and he wanted a governess he should go and take any governess on the register. If he did so, I am afraid his children would have a poor chance of a good education. Surely no person in his senses, or in possession of a sane mind, or whose mind was not affected, would think of taking a governess for his children merely because she was entered on a register as having fulfilled a minimum of qualification. Therefore, the illustration which the gentleman himself takes seems to me to answer itself. It is in truth founded on a mere misapprehension. All you can do by a register is to ascertain qualifications. If you want discrimination, as you do, between governesses and dentists, and between medical men, and between every kind almost of medical practitioners, you must rely on reputation, experience, and knowledge. There is no other resource. You may have as many registers as you like, nay, you may have a compulsory register, but that will not carry you any further, because you must still at last resort, for the purpose of discrimination, to reputation and recommendation; and, therefore, this gentleman's idea that it would be injurious to the public, because according to him the public are so foolish that they would rush and take the first nurse they could find without discriminating between one and the other, is founded upon a misapprehension.

That, my Lords, leads me to ask the question, what will it do? It will assure you at any rate that the person whose name is on the register as having been registered on a certain day, did produce certain credentials, both of character and of competence, to the Registration Board of the British Association of Nurses, and that they were satisfied by the production of those references and the inquiries they made. That, of course, it will deal with, but as between one nurse and another it will tell you no more than you would know by finding two men on the medical register. Then he says, and this is an extraordinary argument, and I am not surprised at all my learned friend read it from the witness's statement (because he would not, I am sure, have liked in so many words to place such a contention before your Lordships), that it would be injurious also to the medical men. Again, I am not sure that I understand the gentleman's meaning, but it is contained in the answer to question 7,771. If I understand it aright, it means this—that if you raise the standard of nurses, and if you make nurses generally more competent, and make the facility for obtaining competent nurses greater, you will supersede the medical man, and you will in time oust the medical man, because you will have merely the physician who is called in now and then, and instead of leaving the case to the general practitioner he will be able to leave it to the nurse, and therefore, it is said, to establish a registration of nurses will be injurious to the medical profession. Your Lordships will observe the assumption that runs through that argument—that registration will so facilitate the employment of qualified nurses as to render them competitors with the medical men. Unless one saw it here, one could hardly believe that such an argument could be used. What he says is this: "Might I now finish my remarks about the relation of this to the medical practitioner? It is this: that nursing is taking an increasing place in medical practice, and a certain number of general practitioners begin to feel that their position is seriously encroached upon, not only to their disadvantage, but to the greater disadvantage of the patients, by the increasing power and position of nurses. If a patient has typhoid fever the argument is, 'Well, there is nothing to be done but feed the patient, and nurse the patient; let us have a consulting physician once or twice a week, and a couple of really well-trained practical nurses,' and the general practitioner would merely come in to look at the tongue and feel the pulse. A number of instances could be brought forward to prove that, greatly to the detriment of the patient and of the practitioner of medicine, nurses have taken the position that should have been held by these gentlemen, and if these nurses have any kind of diploma or any kind of document that can be made use of to indicate that they have passed through a hospital training, have passed certain examinations, and so on, although it is only fair to the nurses to say that in the great majority of instances it would not be unfairly used), it is only reasonable to suppose that it might be unfairly used. They would still further damage the position of a certain number of medical practitioners." Observe, my Lords, the assumption that runs throughout that statement, namely, that the keeping of a register, such as we desire to keep, and such as we do keep, of nurses, will have the effect of so improving their quality, and improving their facilities for their employment, that they will in time become competitors with medical men. I should not require, or ask, for a greater testimony to the value of registration if that argument be well founded. Those are the arguments which my learned friend put forward in answer to my inability to understand the reasons why registration was said to be against the interests of the medical men, or the public, or the nurses themselves. My Lords, it would not be unfair merely in answer to that line of argument which was adopted by my learned friend to say, "Well, but the nurses and the medical men do not say so." I stated to your Lordships the facts, as I was bound to do, as to who the gentlemen and ladies were who supported me, but I am not aware that I ever bragged of the support which I have received. If I did so, I hope that it is entirely contrary to my character, and certainly contrary to my intention.

Sir Richard Webster: I certainly never said anything about your bragging, Sir Horace.

Sir Horace Davey: You did not use that expression, I know, but you can say a thing without using a particular word. When we are told that it is contrary to the interests of medical men and nurses alike, I think it is not otherwise than useful to see what the nurses and medical men think about it. I will not further refer to that subject beyond reminding your Lordships of the documents I referred to the other day, including the letter I read from the *Times* newspaper in answer, be it observed (and I beg my learned friend to notice this), to the circulars which it was said were sent broadcast over the country, although, of course, there was no canvassing at all. Those circulars were headed "private and confidential," but they were sent for the purpose of building up opposition to our petition. The result of all the opinions which my learned friend has read—opinions of various persons who are more or less entitled to express them—is that the register is not a bad thing, but some of them think that the profession is not sufficiently advanced. Some of them say we must wait twenty or thirty years. I believe that is Miss Nightingale's opinion, but through all there runs this persistent misapprehension that it is to be a compulsory register. I agree, if you desire to make it unlawful, to employ a nurse who is not on the register, then totally different considerations will apply. My learned friend may be quite right in saying that a thing of that kind, which deprives a certain number of English people of the opportunity of gaining their livelihood in the way in which they have been accustomed to do, ought to be dealt with only by Act of Parliament. It has been pointed out many times (of course, my friend is conscious of that) that those considerations which apply, and those safeguards which are necessarily applicable, to compulsory registration, that is a register with a prohibition against anybody not on the register practising the profession, have no application to a register such as we propose, which is of a wholly voluntary character. Anybody who likes may come. We ask merely for power to do this. My learned friend calls it a chartered register. He may call it what he likes. He says that certain persons, who profess with more or less warrant to speak on behalf of the Association, have expressed themselves in favour of a compulsory register, and have used language which implied that they hoped or believed that when they had a Royal charter they would have the power of keeping a compulsory register. I know nothing of all this. I appear before your Lordships merely as counsel for the petitioner, whom I named to your Lordships. I take the liberty of saying that whatever respect I may have for Mrs. Bedford Fenwick who, I have no doubt, is an admirable, and disinterested, and public spirited lady, I do not go to Mrs. Bedford Fenwick to inform me as to what the powers and nature of a Royal charter are. I decline to be led into a discussion with my learned friend as to how far Mrs. Bedford Fenwick, or Mrs. anybody else, takes a right view of what would be the position of an association under a Royal charter. I am asking for a charter of incorporation for this Association. I am asking for nothing more than what the charter proposes to give, and if my friend says this, that, or the other will happen, or people will think it means more than it really means, then so much the worse for the people. It cannot affect my application because other people say, or my learned friend may think, that the position of a chartered company is more than it really is, or because other people have thought their compulsory powers would be greater than they really will be. All that is beside the mark, and I was a little bit surprised at the course my learned friend adopted. I will not commit the vulgarity of suggesting that it was in default of a better argument, but it was rather astonishing that he thought it worth while to trouble your Lordships with the so-called evidence which these ladies had given before the Royal Commission for the purpose of gravely telling your Lordships what the effect of the charter was to be.

Lord Hobhouse: I do not think that is quite the case. The charter confers on you no legal powers that you cannot acquire without it, but you ask for it in order to give you greater advantage and greater moral effect, and therefore I think Sir Richard Webster is entitled to refer to the opinions of the promoters to show what is the kind of moral effect that you expect to result from the grant of the charter. That, as I understand, was his object in citing those opinions.

Sir Horace Davey: Then, my Lord, if that is the meaning of it, I have not the slightest objection.

Sir Richard Webster: Your Lordship is quite right.

Sir Horace Davey: I do desire to have the *status* of a chartered body. I say so in the reports my learned friend cited and I say so still. But for what purpose? Not for preventing other people earning their living; not for the purpose of preventing anybody who thinks fit employing nurses with such qualifications as they think fit. I did not know until my learned friend said so that Mrs. Bedford Fenwick was an active promoter of this Association, but it was from my ignorance on the subject that I had not even heard the lady's name. I beg the lady's pardon if she is present in court, but, of course, it was my ignorance and shows that I require to be very much more instructed.

Sir Richard Webster: I think both she and her husband petition in support.

Sir Horace Davey: The petition is the petition of the Princess Christian. I daresay my learned friend is right. Therefore I say it is a misapprehension. You cannot look when powers are asked for beyond what the powers are themselves. If people choose to say that this is a compulsory power I cannot help it. I cannot prevent any amount of misapprehension as to our legal status or legal existence. I repeat that what we do desire is—if my learned friend says so in an authoritative manner I do not object—to keep a register of all people if they think fit; and if they think it will be to their advantage they can enter their names and be published to the world. We do desire that. I do not hesitate to say that that is our desire, and we think rightly or wrongly that it will be of great advantage to those nurses, because they will have an opportunity of advertising, if I may so say their qualifications, which at present does not exist. They will get an opportunity of there being a register in existence to which they can refer people for information as to what their qualifications are, instead of as at present having no unity and no register of the kind. That it cannot provide those moral credentials which my learned friend referred to, and which can only be given by personal knowledge or reputation is perfectly true. Now, my Lords, my learned friend has cited a great many opinions of various persons, and amongst others he has cited the opinion of Miss Nightingale. He has I will not say sheltered himself, but he has ensconced himself, to use a trite quotation with a meaning not its own, *sub magni nominis umbra*. But what is it, I ask, and how did he cite Miss Nightingale's opinion. He cited it from a speech delivered by Mr. Rathbone, who is known to a great many of us.

Sir Richard Webster: It is stated also in the Blue Book and in other passages. That is not the only passage.

Sir Horace Davey: I will read it from your appendix.

Sir Richard Webster: Quite so; that is right. I did read it from Mr. Rathbone's speech, but it is quoted in a great many other places in the Blue Book.

Lord Hobhouse: You also referred to the second report of the Lords Committee at p. 798, but I suppose that is quite sufficient.

Sir Horace Davey: Quite sufficient, but I want to see what it was Miss Nightingale said because it is not otherwise of importance. If you look at what Miss Nightingale is reported to have written to Mr. Rathbone your Lordships will find it at page 798 of the report. My impression was that it was a quotation from a speech of Mr. Rathbone, in which he quotes a letter which he states he had received from Miss Nightingale, and in which Miss Nightingale is reported to have said: "You cannot select the good from the inferior nurses by any test of system of examination. But most of all and first of all, must their moral qualifications be made to stand pre-eminent in estimation. All this can only be secured by the current supervision, tests, or examinations, which they receive in their training school or hospital, not by any examination from a foreign body like that proposed by the British Nurses Association. Indeed, those who came off best in such, would probably be the ready and forward, not the best nurses." Now, my Lords, if my learned friends had intended to rely on that letter, it would have been better if they had brought it to our attention. I will not complain of the letter itself not being printed because it may not have contained anything else beside that passage, and I am quite certain that Mr. Rathbone would not quote it unfairly, but your Lordships will observe that Miss Nightingale's opinion is itself founded on a misapprehension. She expresses an opinion which I have heard a great many other people express, and which is not confined to Miss Nightingale, that examinations are of very little value. It may be true, or it may be false, but it has nothing whatever to do with this matter, because this body, as your Lordships now know by this time, does not purport to be, and is not, an examining body. It apparently had been represented to her that the Association purported to institute an examination for nurses similar to the examination which medical men have to go through, and she expresses her opinion upon that, and expresses her opinion in terms which, so far as I am concerned, I see no reason to dissent from; but the opinion expressed, I repeat, so far as the present inquiry is concerned, is expressed under a complete misapprehension as to the nature of the work which the Association desires to do, and it has nothing whatever to do with any question before your Lordships. Then, my Lords, Mr. Rathbone, to whom we are also indebted for the other opinion expressed by Miss Nightingale, said: "Twenty or thirty years hence, when so much progress has been made that our present time is looked back upon as the time of bad nursing, this arrangement might do." I am obliged to my learned friend, and I am obliged to Mr. Rathbone, and I pay my humble respects to Miss Nightingale. Where I differ from her is in saying we are to wait for twenty or thirty years. Twenty or thirty years hence they will say nursing has not yet reached perfection. Let us wait twenty or thirty years more. In twenty or thirty years nursing will have received still greater development, but again we shall be told it has not yet reached perfection; we must wait till it does. My Lords, I have heard that sort of argument before, and I am quite sure that some of your Lordships who have been engaged on public questions all your lives have heard that sort of argument before.

Lord Hobhouse: I suppose thirty years ago it would have been a good argument. You would not have registered all the "Gamps" of the day.

Sir Horace Davey: No, my Lord, and it is those we want to keep out. I have heard it said, for example—to compare a totally different class of subjects—do not go to the expense of improving your artillery now, because a few years hence your artillery will be perfected to a much larger extent. Do not go to the expense of lighting your towns or houses with electric light, because a few years hence electricity will have received development. If we are to listen to that kind of argument, we should never do anything. If we are to always wait because what we are engaged on is susceptible of greater improvement, we should all stand still and gaze while the men of science and other people were kindly preparing to improve the particular science, or art, or profession in question. My Lords, I must say that I bow with all humility, as your Lordships would, I am sure, do respectfully, to anything Miss Nightingale says on the subject. I am, at least, not likely to forget, nor will, I am sure, any of your Lordships forget, the services which Miss Nightingale has rendered in an unobtrusive and feminine manner, both to the nation and to the profession of nursing; but for all that, my Lords, I am bound, when her opinion is cited in the way my learned friend cited it, to look at it and to see whether that opinion is given under a true estimate of the state of facts; whether that opinion is directed to the existing conditions and the existing state of facts, and also, it is not the slightest want of respect to Miss Nightingale to estimate and weigh, as far as you can, the grounds upon which her opinion is based. Unfortunately, we are often obliged to differ in opinion from those whom we respect, and, of course, no one should presume to differ lightly from an opinion of a competent person; but one of the greatest misfortunes in the world, I repeat, would be if we allowed ourselves to be dominated and allowed our judgment to be ruled by the opinions of persons who presumably are authorities on the subject. Weigh them if you please, but bow to them I, for my part, never will acquiesce in. Now, my Lords, my learned friend cited a number of other opinions, and he said, with perfect truth, I have spoken with respect—and I hope I shall always speak both with respect and courtesy of every person to whom my learned has referred—of Miss Lückes, who, no doubt, has done good work in training nurses, but I am not aware that her opinion is better than that of any other skilled person in the matter. My friend cited the opinion of Mr. Rathbone and Mr. Bonham-Carter, both of them gentlemen whose intelligence and abilities command respect for any opinion which they deliberately express, and I am free to admit that the opinions of those persons are entitled to just as much weight as the opinions of any other intelligent, well-educated gentleman of the highest character and abilities, but no more. Well, my Lords, I will pass, therefore, from the opinions which my learned friend has read in his favour. I forbear to trouble your Lordships with the contrary opinion which has been expressed, because a great deal of this which is called evidence is merely an expression of a gentleman's opinion and a lady's opinion.

Lord Hobhouse: That is always the case.

Sir Horace Davey: I might pick out a great many passages in which opinions are expressed by gentlemen and ladies

—I dare not say as competent as my learned friend's, because I do not wish to differ from him—but, at any rate, who are entitled to express opinions on the other side to that of my learned friend. But my learned friend, although he cited these opinions, did not mention, and it was not necessary for him to do so, because I had mentioned it, but he seemed to leave out of sight that the House of Lords Committee, which was an unusually strong select committee, after hearing all these opinions and what is called evidence, had come to the conclusion I read to your Lordships that three years' training was required to satisfy the exigencies of a thorough training. Now, my Lords, there are one or two points which it is proper that I should say a few words upon, and I desire to say one word at once upon them. My learned friend has criticised, and justly criticised, some language of our charter. My Lords, I had, when I read the charter, very grave doubts in my own mind as to whether any charter ought to contain a power to prescribe penalties by bye-laws. If I were asked —your Lordships will not, of course, care to know much what my opinion would be—but if I were asked, my opinion would be that it would be incompetent for the Crown to give power to any private body to create penalties, and, therefore, on that being pointed out I think it can only have been put into the charter through following a bad precedent. Probably the precedent from which the formal terms of the charter were taken contained it. My Lords, there are bad precedents, but I at once say that probably your Lordships——

Lord Hannen: You do not ask it?

Sir Horace Davey: No, my Lord, I think it would be objectionable. I go further, and I should personally, following my own inclination, ask your Lordships to leave it out, because I think it objectionable that the Crown should give the power to any private persons to inflict penalties, and I think we should all agree about that. There is one other thing about the bye-laws I should like to say. I anticipated that either your Lordships or my learned friends would point out that this charter does not contain a provision which I believe is usually inserted in charters at the present time—it certainly is in other charters of this description which have come before me recently—namely, that the bye-laws should be approved by some body, and that body usually is—my learned friend friend will correct me if I am wrong—the Privy Council itself. In some cases some other body is substituted for special reasons, but I think it is usually the Privy Council, and I think it would be right that this charter should follow the precedent of other charters, and that no bye-law should come into force unless and until it has been approved by the Privy Council.

Lord Hobhouse: In this case it would be the Privy Council. It depends on the special subject matter. It is the Board of Trade sometimes, and sometimes the Local Government Board.

Sir Horace Davey: And sometimes some external body, like the Medical Council. I have known that; but usually it is one of the great Government departments, and unless there is some reason for selecting some other department it is usually the Privy Council, and probably your Lordship would think that right. Now, my Lords, if that is so, it answers a good deal of my learned friend's argument. My learned friend has directed a large portion of his argument to the question whether or not three years is necessary for a qualification, and I cannot help thinking upon that that my learned friend has been a little hard upon us. We are quite able to take care of ourselves, but still he has been a little hard on us, and has been a little inconsistent, because he has endeavoured to show that incompetent persons get on to our register, and has made it a matter of complaint against us that there are persons on our register who have not had three years' training, and on the other hand, he has made a complaint against us that by fixing three years training as the qualification for persons to come on the register we exclude from it persons who enjoy the confidence of these training schools and hospitals for nurses who are, in the opinion of those best qualified to judge, competent nurses, but still would not be able to satisfy the minimum qualification for our register. Your Lordships will at once observe that qualification in the first place is not contained in the charter. It is a matter which is left to be dealt with by bye law. My clients have adopted in the regulations of their unincorporated body the three years' qualification, in pursuance of the report of the House of Lords. That report of the Select Committee of the House of Lords has no coercive or binding effect. It is nothing more than the opinion of a number of competent gentlemen, expressed after hearing the evidence which was laid before them; but still it is the opinion arrived at by a number of gentlemen, who were competent to form an opinion, after having themselves investigated the whole matter and received evidence of the bulk of which, at any rate, no complaint can be made. Therefore, my Lords, my clients, rightly or wrongly, in their regulations, have adopted the three years' training as the average training which should be required to entitle a nurse to be called a trained nurse. Of course my learned friend is perfectly correct in saying that there are people who have a genius for nursing, and who with six months' training are much better nurses than other persons would be in ten years. Of course that is true. There are gentlemen who after six months' study would be better doctors than some of us would be after a lifetime's study, and there are gentlemen, I do not hesitate to say, who would be better lawyers after a year's training than some people would be if they studied law for the whole of their natural lives. Of course there are. There is no doubt about that. Of course, this not being a compulsory register, the hospi als which have such large opportunities, and will continue to have such large opportunities of recommending nurses, will be able to say, "This lady is a perfectly competent and a perfectly skilled nurse; it is fair to say she has only been with us a certain time; we have no hesitation in recommending her, notwithstanding she is not on the register." Of course that will be perfectly open to them, and of course if your Lordships, or whatever body is to approve our bye-laws, consider that a bye-law is objectionable in putting the standard too high, it will be perfectly competent for your Lordships to point that out, but at present the only thing approaching I will not say a judicial, but any authoritative resolution or finding upon the point is the report of the Select Committee of the House of Lords. It is not proposed to make that part of the charter. It will properly become part of the regulations.

Then, my Lords, my learned friend said that our register is incorrect. My Lords, I am far from saying it is perfect. Indeed, I am a long way from saying it is perfect; but I may perhaps be permitted to say to my learned friend's clients through him that it will be more perfect if they would give us their assistance, and answer our applications for information regarding the nurses. I am glad to say that I make an exception of King's College at any rate, which is one of my strongest opponents; but King's College does answer our letters and does give us such information, and I have no doubt all the information they possess, and if the other training schools will follow the same example, I think it would be very largely conducive to the improvement of the register. Now, my Lords, I followed my learned friend as far as I was able in the book he was kind enough to lend me, which had been numbered, but it seemed to me that his instances came to very little indeed. He cited an instance, to take one, of a nurse stated to have been in Guy's Hospital, 1872-73, and said that the lady in question was only there for a limited time. But it does not say how long she was there—1872 to 1873. It then says that she was in the Wimpole Street Institution from 1873 to 1875, and in the North Audley Street Institution from 1875 to 1883, and is a member of the London Association of Nurses—that is not our Association—to date. My learned friend does not say it is inaccurate, but he says it does not say the exact time during which she was in Guy's Hospital. Well, my Lords, I am very sorry, but in the limits of a register of this kind it would be surely impossible to give that information. It does, at any rate, direct the attention of those who thought of employing this particular lady to the institutions where they can learn more about her than they can from this register. Then there is the same thing about the Royal Free Hospital and the earlier ones. Then my learned friend said that upwards of six hundred have no hospital qualification, and there is an absence of any qualification. Of course that depends upon what my learned friend means by hospital qualification. I understood from the reference he made that he would not consider the Leeds Infirmary, 1880, any hospital qualification, and there were some other institutions which I gathered he would not consider hospitals. Well, my Lords, that is of course a question of words, but at any rate, whatever it is, it contains no misrepresentation. It does not tell people they are trained nurses when they are only probationers, and it does not advertise to the world that we will send out trained nurse supplies at short notice, and then send out probationers who have never been out before. That is not there. But at any rate it tells the public fairly when and at what time and so far as can be done in a condensed form of this kind, during what years the nurse in question was at the institution, and what institution she was at. I am told, my Lords, by the gentlemen who instruct me—they, of course, had not their attention directed to this till my learned friend stated the figures on his instructions—that my learned friend has exaggerated the number, but probably the meaning of that would be that some cases which he would consider no hospital qualification other people would treat as a qualification. But, my Lords, my learned friend forgot this passage in the preface, which tells you that during a certain time and up to a certain period, any person who had in fact nursed for three years, whether trained in a hospital or not, but had been engaged for three or more years in nursing the sick, provided their credentials and testimonials were satisfactory to the eminent persons who formed the registration board, were put on the register, which would of course account for the large number of persons being on the register who would avail themselves of that liberty, though they had not been trained at a hospital. That is in entire accordance with previous precedent. I looked, for instance, at the Dentists' Act, and I find that when the dentists' register was formed those persons who had been *de facto* practising dentistry for a certain period before the date of the Act were allowed to put their names on the register, stating the number of years they had been engaged, and you must commence the register in that way, taking care, of course, that after a certain period of grace, as it is called, the proper qualification shall be affixed. So that it is quite possible—I do not know that it is so, because there would have to be an examination in each individual case—that many of those names to which my learned friend referred were names of young women who had been put on the register, having satisfied the registration board of their character, and that they had *de facto* been engaged in nursing the sick during the period of three years. Then my learned friend says: "Oh, but if you put at the head of your register that you do not answer for the competency of your nurses, there would be an end of your register." My Lord, my learned friend did not observe that that is exactly what we do. We do put at the head of our register in the preface of the book which contains the register, and on the page immediately preceding the register: "In accordance with the suggestion made by the Select Committee of the House of Lords appointed to inquire into the Metropolitan Hospitals, the Registration Board wishes most distinctly to state that it cannot answer for the technical knowledge of any registered nurse, nor for the nature of the training which she has received, but that every hospital must of necessity be held responsible for the credentials which it has issued, and upon which the Registration Board is compelled to rely." That seems to be a perfectly fair statement, and if my learned friend says that our register would be rendered valueless if we put at the head of it that we did not answer for the competency, my answer is that we commit that crime already. We do put at the head of our register that we cannot be answerable for the technical competence or knowledge of any nurse; that those hospitals who give certificates, or who give registration, must be answerable themselves. If they do not desire by their certificate to have their certificate treated as a testimonial of competence, but merely a record of the fact that the person has been in the hospital for a certain period of time, let them frame their certificates in any way they please. That is perfectly open to them, or in any proper case where they cannot honestly give a certificate of competence, let them withhold the certificate altogether. But if they do give certificates, and certificates in one form which apply to those who are most competent, as well as to those who are least competent, they cannot complain if the public are obliged to rely upon those certificates which they give. My Lord, my learned friend has said, and said with perfect truth, that there is a great difference in nurses. I will not dwell any more on that point, because upon that there will be no difference of opinion between us. Then my learned friend read Mrs. Bedford Fenwick's view of the Royal charter, and as to what she anticipated, hoped, and wished would be the ultimate effect of establishing a register. Mrs. Bedford Fenwick may be sanguine, but I take the liberty to say to her, or to any ladies who may have the same sanguine anticipations as that lady indulged in before the Select Committee, that if they desire any powers of that kind they must not content themselves with the humble means afforded by incorporation under charter or Board of Trade licence, but they must seek for and obtain an Act of Parliament. Of course no such compulsory powers could be exercised, and it would not be right that anybody should be authorised by the Queen to deprive other people of exercising their profession by imposing a condition on their exercising it; indeed, I doubt whether such a charter would be good. I have a strong suspicion that the charter would be bad as being a restraint upon trade; but, at any rate, whatever are the anticipations, hopes and wishes, and sanguine expectations of those ladies, I am afraid they will have to wait till they obtain an Act of Parliament, which may or may not be obtained hereafter. Now, my Lords, your Lordships have heard that competent nurses are excluded from our register. I am afraid that that must be said of every register or every list which prescribes a minimum qualification. There are, of course, geniuses, or, without being geniuses, there are persons who, by skill and industry and native aptitude, become far more competent after a few months', or even a few weeks' training, than other persons will become after a training of many years. But, my Lords, we cannot help that. All we can do is to invite persons who do fulfil certain qualifications to register themselves. Persons will be perfectly justified in stating the reasons why their names are not to be found on the register. Nay more, if it should be found by experience that a large number of competent nurses are not on the register, it will create such discredit to our register as will justify the world in disregarding our register so much that it will cease to be of any value, either as a credential or anything else; and for mere preservation sake it is not likely that the Association, by their bye-laws and regulations, approved of, as they will have to be, by the Privy Council, will prescribe such conditions as would stultify themselves and render their register useless. My learned friend seems to be difficult to please. On the one hand, he complains that we exclude from our register a number of competent persons; and, on the other hand, that we include a number of incompetent persons. He says that we admit persons not having three years' training, and, on the other hand, he complains of three years being fixed, because it excludes persons who have not had that length of training and yet are competent nurses. My learned friend cannot have it both ways. But, my Lords, my answer to both accusations is that that is a matter of regulation. We must assume in fairness in this Association, as well as in every other body of ladies and gentlemen, that they will exercise their powers so as to attain, and not to defeat, their object; and for that purpose that they will neither put this qualification so high as to exclude the competent, nor will they put this qualification so low as to include the incompetent. Of course I speak of an average. You cannot do more than that. Many of the objections my friend hinted at and put forward were really objections that will equally apply to every register or list kept in the world. Then, my Lords, my learned friend commented on the difficulty of getting a person off the register when once on. Of course that is a difficulty. There is a natural reluctance to giving information which may have the result of depriving a woman from earning her living. That feeling is what we all have, and I am afraid we cannot get rid of it; but we do afford a body to which complaints may be brought, whereas at present, unless you prosecute the woman, unless she has committed some indictable offence and is prosecuted, you have no means whatever of preventing her following the profession of Mrs. Gamp. The hospitals cannot withdraw their certificates. Their certificates are true because they certify that she was there for a certain time, and gave satisfaction during that time. Therefore there is no means by which a person, short of being convicted and sentenced to imprisonment, can be struck off, and not even then always, because you cannot prevent dishonest persons and bad characters from palming themselves off on the public and obtaining custom in that way. You want a body to which you can go and make your complaint, and if a woman is on the register, then there is such a body in existence.

Lord Hannen: How would they take her name off? Your charter does not indicate it.

Sir Horace Davey: Yes, my Lord. The charter gives the power. Your Lordship will find it on page 7. It provides for the person in question being heard, and that the General Council shall meet at such times as may be prescribed by the bye-laws, and that the ultimate decision on any matter affecting the corporation shall rest with the General Council, and the General Council shall have power to expel.

Lord Hannen: That is from the corporation.

Sir Horace Davey: Yes, my Lord. "To expel from the corporation or suspend from membership any member who shall, after full inquiry, and after hearing such member, be deemed by the Council unworthy to remain a member or to act as such by reason either of moral delinquency or professional incompetence, provided always that no member be so expelled or suspended at any meeting of the Council unless twenty-five members of the Council at the least be present at such meeting, and unless not less than three-fourths of those present and voting shall vote for such expulsion or suspension, and provided further that any member so expelled or suspended shall have the right to demand that before such expulsion or suspension takes place the matter shall be referred for decision to a general meeting of the members of the corporation to be forthwith summoned for the purpose." Your Lordship is quite right. There is power in the regulations.

Lord Hobhouse: You could take people off the list, I suppose, because nobody has a right to be on?

Sir Horace Davey: Yes, my Lord; there is a power in the regulations.

Lord Hannen: But those regulations are no part of the charter.

Lord Hobhouse: Except members of the corporation no one would have a right to be on, as I understand. The others would be volunteers.

Lord Hannen: But I understood that any nurse would be competent to apply to have her name put on the list although she was not a member of the Association.

Sir Richard Webster: A number of these people are not members.

Lord Hannen: She being on the list, where is the power to take her off?

Lord Hobhouse: You cannot strike her name out of the published list.

Lord Hannen: I do not find that there is any provision to that effect.

Sir Horace Davey: That is in the regulations, my Lord.

Lord Hannen: But they are not incorporated with the charter.

Sir Horace Davey: That is so. There is an actual power in the bye-laws to expel any member after a hearing.

Lord Hannen: I think it might be necessary to make it a condition of persons being admitted, and having their names on the list, that they should be subject to removal.

Sir Horace Davey: That is referred to, my Lord, on page 16 of our case. Your Lordships will see there the conditions which are necessary for registration—"No nurse shall be registered until he or she has produced such proof to the Board of professional education and moral character as the General Council shall from time to time require, nor unless application for registration is made upon a special form supplied for that purpose. The Registration Board shall have the power to direct the Registrar to erase from the register of trained nurses the name of any nurse who shall, after full inquiry, appear to a majority of two-thirds of a meeting of the Board to be unworthy to remain thereon. But no name shall be erased from this cause except by order of a meeting of the Board specially summoned to consider the matter, and at this meeting fifteen shall be the necessary quorum."

Lord Hannen: Yes; that seems to provide for it.

Sir Horace Davey: "Provided always that any nurse whose name it is proposed to erase shall have the right to appear in person, or by proxy, before the Board, to show cause why such erasure should not take place, and shall, moreover, have the right to demand that, before the name is removed from the register, the matter shall be referred to a meeting of the General Council." That is made part of the contract by being printed in front of the application.

Lord Oxenbridge: Has that been agreed to by all the nurses that form this Association?

Sir Horace Davey: It is on the application form. Some of them, I believe, came in before the application form was sent out.

Sir Richard Webster: I am told that that is not the original one.

Sir Horace Davey: There may have been some admitted before that application form was used, but all those who have come in since that form of application was used are bound by it.

Lord Oxenbridge: When did this particular form come into existence?

Sir Horace Davey: I am told that everyone has signed it. The gentleman who instructs me says that the form containing that regulation printed in it has been used from the very commencement. I think your Lordships will agree with me that that is a proper matter for bye-laws and regulations. That would obviously be right. It might be necessary, according to experience, to alter the bye-laws from time to time, and I can conceive that if a nurse refused to give her present address, or persistently refused to give information as to her whereabouts, a case might very likely arise where the Board had some suspicion, and it might be necessary to make enquiries of the nurse, and if she did not give a satisfactory account of herself, or would not disclose her address, or where she had been employed, they would have a right to say that she should have no longer the advantage of being on the register. It would be possible to extend it in that way. What I mean is, that the particular conditions of a person remaining on, or being removed from, the register may vary, and it is expedient that there should be a certain amount of elasticity about it, and for that purpose that should be governed by regulations and bye-laws, instead of being crystallised in the form of the provision of the charter. However, that is really a matter of detail. Whether they should be in the charter or be dependent on regulations to be approved of by your Lordships, I am quite sure your Lordships will not sanction anything unfair to the nurses themselves, and I shall not say any more about that. Then, my Lords, my learned friend said that you cannot revise the register. I am afraid no register can be ever effectually revised. It has recently been held in the Law Courts that a person once on the register ot dentists, although he loses his qualification, cannot be got off. That is the result of registration. When once you have admitted a person on the register you cannot strike him off except for good cause shown. It would be highly unjust that you should do so. Of course, it is one thing to say that So-and-so is not on the register, and there may be several causes why she is not. She may not herself desire to be, or her friends may not wish it for her. She may be one of those to whom my learned friend referred, who are superior to any registration; but when once you have put a woman on the register it would be the height of injustice to strike her off for anything but good cause shown. It is one thing, for instance, for a person not to be elected a member of a club—that, for various reasons, may happen to any one of us, but it is a totally different thing when, after once being a member of a club, a man is expelled from it. With regard to the profession of nurses, it would be a very different thing to say that So-and-so is not on the register, from saying that So-and so was on the register, but was removed when the register was revised. I must say I think an arbitrary power of revising would, or might, work great injustice among nurses from the point of view I have named, and the utmost you can do with any list or register of this sort, which is to have publicity, is, as far as you can, when cause is shown, to revise it, or it may be even without cause shown, because I can quite understand that there might be a case—although I do not say our rules provide for it—where the suspicion would be so strong, and the answers given by the nurses so unsatisfactory, as to justify the Board in so acting. But, my Lords, more than that we cannot do, and in my humble judgment it would be extremely unjust that the Board should have the power of revising the register in that way. But I should like to ask, when my learned friend says we cannot revise the register and remove people from it when they are once on, what is the present system now? Is what we propose an improvement on what is the system now? My learned friend, although he said a good many things, did not say very much about the register, which the colleges and training schools keep. He did not read the evidence about it in the report, and for a very good reason. Of course, there are so-called lists or books prepared, and I observe they are generally called "black books" in the evidence, but, as far as can be discovered, they are not published, and are not accessible to the public. That is a good reason why my friend would not say much about it; but nobody pretends that those lists, or registers, would tell any more than the facts about the nurse while she was at the hospital, or after leaving the hospital, except in those cases (and they are comparatively few) where the nurses remain, not part of the hospital staff, but among those who have employment in the hospital.

The Marquis of Ripon: The hospital certificate once given is never cancelled or withdrawn, is it?

Sir HORACE DAVEY: Never, my Lord; but cases have not been unknown where very unfair use has been made of them. I will not trouble your Lordship with the names or particulars, but I have cases which I was asked to mention.

Sir RICHARD WEBSTER: In some cases they have to be brought back to be revised.

Sir HORACE DAVEY: But you cannot revise a certificate. The certificate states a fact.

Lord HOBHOUSE: Most prudent people do not accept an old certificate.

Sir HORACE DAVEY: No, my Lord. Then, my Lords, my learned friend, Sir Richard Webster, wound up with an eloquent appeal to your Lordships to pay attention to those who had been concerned in the training of nurses. I should be extremely sorry if it should be supposed that I had ever said, or hinted, or had been instructed to say, or hint anything disrespectful, or that did not adequately appreciate the labours of those who instruct my learned friend; but your Lordships will observe that my learned friend avowedly represents the interests of, or is supported by, persons connected with the training hospitals and schools, whereas the ladies and gentlemen who support the petitioner in the application which she makes, are medical men practising, in many instances, as consulting surgeons or physicians at the great London hospitals, but in most cases having no direct professional connection with the hospitals, and the nurses are nurses who have appreciated the advantage of such a register as we have established at present. Without in the least degree denying that those whom my friend represents are entitled to express their opinions, and I feel quite certain your Lordships will pay due weight to their opinions, still after all you have on our side in substance the medical men and the nurses, and on the other side you have the training schools. It is perfectly natural, and in accordance with what one would anticipate from one's knowledge of human nature, that the training colleges, good work as they have done, and good work as they will do, I hope and believe, in the future, may still be so wedded to their own associations as to think that nobody can advantageously alter them, or that it would be much better if nobody would look anywhere for a nurse except to the training schools and colleges. My Lords, it must not be forgotten with regard to the great hospitals that a large portion of their income is derived from their sending out nurses, who, as was alleged before the Commission, were not perfectly trained nurses, to houses and receiving payment for so doing. Therefore, it is perfectly natural, and only in accordance with what one would anticipate, that they should dislike any third body which presumed or dared to keep a register of nurses, and dared to tell the world that any nurse had any qualification except their own. That feeling, no doubt, is somewhat intensified in the case of some of the hospitals (but not all I agree) by the fact that a large portion of their income depends upon their supplying nurses, and it is possible that if those nurses were not, as they would probably be, upon the register, it might to a certain extent, perhaps to a large extent, interfere with their income. That persons are directly influenced by motives of that kind may not be the case, and it may be that many of the ladies and gentlemen sitting around my friend would at once disclaim any mercenary motives, even on behalf of a hospital, and although it may be vicarious; but none of us, not even your Lordships, can tell the motives that impel you to a particular course of action. I do not suggest, of course, that it is for any pecuniary interest, but it may be that the pecuniary interest of the institution in which they take an interest, and whose success they properly and wisely and disinterestedly desire should have some weight in determining their course of action. On the other hand, many medical men—I am not going to repeat their names or numbers—who are not the least distinguished by reputation, think that the establishment of some kind of registration of this kind, voluntary in its aim, is essential for the protection of the public and the medical profession, and surely those men have a right to be listened to, and they can form a better opinion, I was going to say, upon the effect on the medical profession and on the public at large, even than those whose experience is derived entirely from the training schools and the hospitals. They live in the world, and not in the world of the hospitals, or the training schools, and they know what the wants of the medical men are. I venture to submit that their opinion is entitled to at least as much weight as that of the eminent and disinterested persons whose views my learned friend has placed before your Lordships. Nor will I say much about the nurses themselves. I have told your Lordships the number of nurses I represent. I agree it was my fault, and I made a mistake in the figures as regards the number of nurses on the register. I should have said rather the number of nurses who are members of the Association. I am obliged to my learned friend for correcting that statement, but at the same time the number I gave does represent the number of persons who have become members of this Association, although they are not all on the register. It is idle to talk about what is for the benefit of the medical men and the nurses. Surely it is much better for the medical men and the nurses to speak for themselves. Of course my learned friend is entitled to say that the best of the nurses are those in the hospitals. But, my Lords, that is an argument I have heard before. In other words it is this: that when persons have only got a minority they always say our minority contains the best of the voters.

Sir RICHARD WEBSTER: No.

Sir HORACE DAVEY: My learned friend says, in effect, that his minority is of a better quality than my majority. My Lords, I am not disposed to admit that at all. I press upon your Lordships that according to the figures given here the number of nurses supporting the incorporation are 3,700, whereas those petitioning against it are 540 only.

Sir RICHARD WEBSTER: That includes the whole of the names of the members of the Association who have supported it personally, but have been signed for by the President. We have the names of those who have petitioned against it.

Sir HORACE DAVEY: Some of those nurses who have availed themselves of the register who are still nurses in the London Hospital or St. Thomas's Hospital have been induced to sign the petition against it. That is quite likely. My friend has not denied that the bulk of the trained nurses of this country, estimated not by us, but by other people, to be 5,000, do desire that these powers should be conferred upon this body. My Lords, I have not heard one word of argument to alter that. Further than that, the training colleges are by no means unanimous, because a large number are in our favour, and it is remarkable that those institutions which give the longest training are those most favourable to the proposal which we make. St. Bartholomew's Hospital, Middlesex Hospital, University College Hospital, St. George's Hospital, the Metropolitan Hospital, the Royal Free Hospital to a certain extent, Guy's Hospital, and Charing Cross Hospital. So that, my Lords, even among the training schools and hospitals there is by no means unanimity, and it is a remarkable coincidence that the dividing line seems to be where the most training is necessary for the pupils. My Lords, I venture to submit that the prayer of this petition is a moderate prayer, and one which is conceived in the interests of a class of the community who are entitled to special consideration, and if your Lordships can see that the views that have been expressed, which are printed in the papers before you by the medical men, and by the nurses, and which have been feebly and inadequately expressed by their counsel at your Lordships' Bar, are worthy of consideration, I pray that you will advise Her Majesty that this charter of incorporation, according to the draft, with such alterations as I have suggested, and others your Lordships may think it right to introduce, may be granted to this body, notwithstanding the arguments which have been addressed to your Lordships by my learned friend Sir Richard Webster, in opposition. My Lords, I would only say in conclusion, what I said at the beginning, that I do not conceive, as regards the mere grant of the charter, that there is really any difference of opinion between my learned friend and myself. It is only a question whether the charter should assume the particular character which we propose so far as its purposes are considered; and as to that, I point out to your Lordships that my learned friend's clients have not the courage of their opinions, because if they had the courage of their opinions they would say, not strike that purpose out, but put in a prohibition against issuing any such list or register. Suppose your Lordships struck the purpose out, still we might go on registering, and we might say the British Nurses' Association keep a register of trained nurses. There would be no illegality in that, and my learned friend can hardly dispute that. Their argument ought to have led them to ask not only that the purpose might be omitted, but that there might be a prohibition against this chartered association, as I hope it may one day call itself, keeping or publishing any register of the kind, so as to make it unlawful for them to do it.

Lord Hannen : What do you say to the objection made by Sir Richard Webster that your register purports to be of trained nurses generally ?

Sir Horace Davey : My Lord, I should have thought, reading it fairly, that was not the meaning of it. It does not purport to have any coercive or any compulsory effect. It is headed "The Royal British Nurses' Association. Register of Trained Nurses for 1892." That is to say, it is a register of trained nurses kept by the British Nurses' Association for the year 1892.

The Marquis of Ripon : It would rather give the preference to the register of trained nurses.

Lord Hobhouse : Your charter provides for keeping a register of nurses indefinitely ?

Sir Horace Davey : Yes, my Lord. I do not know where my learned friend got the word "directory" from, which he frequently used. I say it is not merely a directory containing names and addresses, but it is the maintenance of a list or register of nurses, showing as to each nurse registered her name and address, and the name of the hospital and other places at which she has been trained, and the length of training she has received. That is one of the purposes for which we desire incorporation. We desire to keep, maintain, and publish a list or register of nurses; there is nothing coercive about it, because no human beings need put their names on it unless they like, and it is not a condition precedent to employment by anybody that the name should be found there. All we desire is, as one of our purposes, to keep a list or register of this description, upon which any nurse who thinks fit, and complies with the requirements, may have her name entered.

Lord Hannen : Do you object to its being made quite clear that you only purport to keep a register of nurses who have applied to have their names put on the list?

Sir Horace Davey : No, my Lord, I should think not. If it is not clear already it should be made clear, because it is only the fact. I cannot conceive that there can be any objection to that. I admit that a certain number of ladies, if I may say so very respectfully, have talked, I was going to say, a good deal of nonsense ; but, at all events, they have exaggerated the effect of this register a great deal, and I don't think the exaggeration is confined entirely to our side. I have no objection to do anything that will more clearly bring out the truth ; indeed, not only have I no objection to it, but if it is not sufficiently clear, I think it would be right that it should be made clear.

Lord Hannen : There is one other question I should like to ask you. You have said what you have to say about the penalties ?

Sir Horace Davey : Yes, my Lord.

Lord Hannen : What has been the usual form of approval required by the Privy Council?

Sir Richard Webster : As to charters does your Lordship mean ?

Lord Hannen : As to bye-laws ?

Sir Richard Webster : The usual form, if I may be allowed to answer your Lordship, would be that the purposes of the bye-laws have to be stated in the charter, and the bye-laws are not to be enforced until approved by her Majesty in Council. If I may be allowed, I should like to say that my learned friend has not dealt with the governing body, which does not include lay members.

Lord Hannen : Is there anything to show an honorary member would not be ?

Sir Richard Webster : Honorary members, my Lord, have none of the powers of voting under the charter, and the members are to be limited to medical men and nurses only.

Sir Horace Davey : We think that the medical men and the nurses are quite capable of doing their own business.

Lord Hannen : Why do you attach importance to that ?

Sir Horace Davey : I do not, my Lord, but my learned friend does.

Lord Hannen : I should not have thought that the description of members as honorary members would exclude them from the rights of membership.

Sir Richard Webster : I am not raising, my Lord, a technical point at all. It is stated in their petition that their scheme is that the lay board should not have any right as members, and they claim it should only be the medical men and the nurses, and, therefore, in framing the bye-laws, they intend there should be nobody else having the rights of members. It is really a question of substance.

Lord Hannen : Would you object to removing the word "honorary," so that there should be a power to elect sufficiently-qualified persons as members of the corporation?

Sir Richard Webster : Or that they should be there compulsorily.

Sir Horace Davey : I may say at once that this is intended to be a professional body. Of course it may be right that there should be a certain number of lay members.

Lord Hannen : Take an instance. Why should not there be a power for a lady like Miss Nightingale to be a member ?

Sir Richard Webster : I mentioned no names before, but there is Mr. Rathbone, and I may say that on every one of the managing committees of the training schools there have been people like Mr. Gibbs and others.

Sir Horace Davey : We wish, of course, that it should be a professional body ; but if there be any persons, like Miss Nightingale, connected with the profession, by all means let them be on ; but we wish it to be a professional body, and not a popular body, to be governed by Mr. Rathbone and other gentlemen like him. It is intended to be as much a professional body as the Institute of Civil Engineers or the Institute of Surveyors, or the Institute of Architects. I do not see myself anything to exclude honorary members, but I state frankly that we don't wish to be governed by the lay element. I do not see that there is anything to exclude an honorary member from the General Council by the clause dealing with that body.

Lord Hobhouse : Yes, it refers to elected members, and the elected members are a separate class of the corporators.

The Marquis of Ripon : The proposal simply is to take out the word "honorary" from the 4th qualification on page 6. You would then have it in your own hands to make your own bye-laws, but you would also have the power of extending your members.

Sir Horace Davey : I do not object to that, my Lord.

Lord Hobhouse : Then you would have to omit the word "elected" from the qualification.

Sir Horace Davey : Yes, my Lord. It is, I think, a small point, but we wish it to be understood that the view of the petitioner, and those who are behind the petitioner, is that this ought to be a professional body—it is not for the purpose of training. Those gentlemen to whom my learned friend, Sir Richard Webster, has referred may be very useful as advisers for the purpose of training, although I should have thought myself that Mr. Rathbone's abilities lie rather in a different direction. They may be useful advisers for the purpose of considering the method of training nurses for aught I know to the contrary, but in a purely professional body like this, which is intended to be a body of nurses, the members of which are nurses or employers of nurses—I mean the medical men who are associated with nurses—it is desired that the control of the Association should be in their hands. No doubt matrons, for instance, might be made honorary members. If your Lordships think it right to leave out the word "honorary," and to make any member, whether elected or admitted under that clause, eligible for the General Council I shall raise no objection, because it seems to me it would not make any substantial difference in the matter. With those observations I leave the case with your Lordships, humbly submitting that we have made out a case for the grant of a charter as we suggest.

Their Lordships intimated that they would consider the matter and advise Her Majesty.

### Notes and Queries.

### Queries.

(32) *Midwifery.*—Where and how can I train in Midwifery ? Is there an advantage in possessing the L.O.S. diploma ? Where can I train in the North of England ?—*Lancashire Nurse.*

### Answers.

(32) *Midwifery* (*Lancashire Nurse*).—You could apply to Liverpool Lying-in Hospital, and go either as a resident pupil midwife for twenty guineas, which includes lectures, examination, board, and residence, but not washing or uniform ; or as a non-resident pupil for seven guineas, which fee includes lectures, examination, and lodging, without board, at the house of the district midwife. The Manchester Maternity Hospital, Matron Mrs. Wilkie, is another hospital to which you could apply. Wherever you go you must make the London Obstetrical Society's diploma your goal ; without it nowadays you will be nowhere. Write again if these prove impossible for you.

*** In consequence of great pressure on our space this week, we are compelled to hold over till next week a letter from Miss Ritchie on the Clapham Maternity Hospital, and several other papers.

